# Revealing the Complexity in CD8 T Cell Responses to Infection in Inbred C57B/6 versus Outbred Swiss Mice

**DOI:** 10.3389/fimmu.2017.01527

**Published:** 2017-11-22

**Authors:** Matthew D. Martin, Derek B. Danahy, Stacey M. Hartwig, John T. Harty, Vladimir P. Badovinac

**Affiliations:** ^1^Department of Pathology, Carver College of Medicine, University of Iowa, Iowa City, IA, United States; ^2^Interdisciplinary Graduate Program in Immunology, Carver College of Medicine, University of Iowa, Iowa City, IA, United States; ^3^Department of Microbiology, Carver College of Medicine, University of Iowa, Iowa City, IA, United States

**Keywords:** CD8 T cells, immunologic memory, outbred mice, memory phenotypes, protection against re-infection

## Abstract

Recent work has suggested that current mouse models may underrepresent the complexity of human immune responses. While most mouse immunology studies utilize inbred mouse strains, it is unclear if conclusions drawn from inbred mice can be extended to all mouse strains or generalized to humans. We recently described a “surrogate activation marker” approach that could be used to track polyclonal CD8 T cell responses in inbred and outbred mice and noted substantial discord in the magnitude and kinetics of CD8 T cell responses in individual outbred mice following infection. However, how the memory CD8 T cell response develops following infection and the correlates of memory CD8 T cell-mediated protection against re-infection in outbred mice remains unknown. In this study, we investigated development of pathogen-specific memory CD8 T cell responses in inbred C57B/6 and outbred National Institutes of Health Swiss mice following lymphocytic choriomeningitis virus or *L. monocytogenes* infection. Interestingly, the size of the memory CD8 T cell pool generated and rate of phenotypic progression was considerably more variable in individual outbred compared to inbred mice. Importantly, while prior infection provided both inbred and outbred cohorts of mice with protection against re-infection that was dependent on the dose of primary infection, levels of memory CD8 T cells generated and degree of protection against re-infection did not correlate with primary infection dose in all outbred mice. While variation in CD8 T cell responses to infection is not entirely surprising due to the genetic diversity present, analysis of infection-induced immunity in outbred hosts may reveal hidden complexity in CD8 T cell responses in genetically diverse populations and might help us further bridge the gap between mouse and human studies.

## Introduction

Much of our current understanding of immunology has been learned through the study of laboratory mice, and most of these studies have utilized one or two inbred mouse strains. There are many advantages to the use of inbred mouse strains for studying T cell-mediated immunity in health and disease states. Knowledge of host MHC restriction, which is identical in individual inbred mice, has facilitated the development of tools including peptide-MHC tetramers and transgenic T cells to track antigen (Ag)-specific responses. Additionally, genetic homozygosity allows for empirical analysis of how individual genes impact the immune response. These tools developed for use in inbred mice allow for detailed analyses of the immune response in the setting of autoimmunity, following infection and/or vaccination, and in response to tumors, and have provided valuable information for the development of therapeutic interventions during disease states. In most instances, analyses of this kind would be technically and ethically impossible to conduct in humans. Nonetheless, some have argued that mouse models poorly reflect aspects of the human immune system and have suggested that these differences may be to blame for the inability to translate therapeutic treatments described in the laboratory to successful outcomes in the clinic (1–6). Because of this, recent work has sought to devise mouse models that more accurately reflect the status of the human immune system. This work has suggested that the environment in which laboratory mice are housed and their exposure to infections that humans are naturally exposed to can impact the composition of the immune system, and that mouse models that include microbial exposure could be used as a tool to study immunological responses in free-living organisms such as humans (7, 8). While inclusion of multiple pathogen exposures will certainly inform our understanding of the immune system, it is still unclear whether the knowledge that we have gained utilizing a limited number of inbred mouse strains holds true for all mouse strains or can be generalized to humans.

One major difference between humans and inbred mice is that humans are genetically diverse. In this regard, genetically diverse outbred mouse strains are available for scientific use, the most commonly utilized being Swiss mice. Swiss mouse colonies originated from nine mice brought to the United States from Switzerland in the early 1900s, and evidence suggests that the genetic diversity within Swiss mouse colonies is similar to that seen in feral mice and within the human population (9). However, studying the immune response in a genetically heterogeneous population such as Swiss mice presents a number of challenges. Many tools that have been developed to allow for the study of Ag-specific T cell responses in inbred mice are unavailable for use in outbred mice due to unique MHC restriction within individual mice and the inability to perform adoptive T cell transfers due to rejection in incompatible hosts. However, our laboratory has recently described a “surrogate activation marker” approach to distinguish naïve (CD8^hi^/CD11a^lo^) from pathogen-specific (CD8^lo^/CD11a^hi^) CD8 T cell populations in any mouse strain after various types of infections without *a priori* knowledge of their MHC restriction or Ag specificity (10–12). In this model, CD8^lo^/CD11a^hi^ cells represent Ag-experienced cells, as this population expands following infection, but not in response to inflammation alone. Using this approach, we described that magnitude and kinetics of CD8 T cell responses following infection were discordant in individual outbred mice, an observation that was also noted in the current study. However, how memory CD8 T cell responses develop, and the protective capacity of memory CD8 T cells generated following infection in individual outbred mice remained unclear. When we examined these questions in the current study, we found that interestingly, like the magnitude of CD8 T cell responses, the rate of phenotypic progression of the memory CD8 T cell population is highly variable in individual outbred mice, which could impact protection provided against re-infection. Furthermore, the protective capacity of memory CD8 T cells against re-infection did not correlate with the size of the memory CD8 T cell response in every individual outbred mouse. These novel findings suggest a hidden complexity in CD8 T cell responses in outbred organisms, such as humans, that is not reflected in inbred mouse models. Additionally, this study further advances use of the surrogate activation marker approach for tracking CD8 T cell responses in any mouse strain, including strains such as those within the collaborative cross, which could be used in the future to interrogate underlying genetic causes of variability in CD8 T cell responses and CD8 T cell-mediated protection against re-infection.

## Materials and Methods

### Mice, Bacteria, and Viruses

Female C57B/6 and National Institutes of Health (NIH) Swiss mice were obtained from Charles River Laboratories. All mice were housed under pathogen-free conditions and used at 6–10 weeks of age. For co-housing experiments, one to two female C57B/6 mice were housed with three to four female NIH Swiss mice that were 6 weeks of age for 3 weeks prior to infection. The Armstrong strain of lymphocytic choriomeningitis virus (LCMV), attenuated *actA*-deficient *Listeria monocytogenes* (Att LM), and virulent *Listeria monocytogenes* (Vir LM) strain 1043S were grown and quantified as previously described (13, 14). All LCMV infections were administered intraperitoneally (i.p.) with 2 × 10^5^ plaque forming units (PFU). All *Listeria monocytogenes* infections were administered (intravenously) i.v. 1 × 10^4^ or 5 × 10^6^ colony forming units (CFUs) of Att LM were administered for primary (1°) infections, and 5 × 10^6^ CFUs of Att LM were administered for secondary (2°) infections. 1 × 10^5^ CFUs of Vir LM were administered for challenge infections. For all infections, one mouse per cage was left uninfected, and percentage of CD11a^hi^/CD8^lo^ cells was determined periodically to verify that mice were not experiencing unintended infections. All mice were housed at the University of Iowa under the appropriate biosafety level according to the University of Iowa Animal Care and Use Committee and NIH guidelines.

### Detection of Ag-Experienced CD8 T Cells and Surface Marker Expression

Blood was collected *via* retro-orbital puncture and red blood cells were lysed with ACK. For detection of cells in tissues, spleens, and inguinal lymph nodes were collected, and tissue was processed into single-cell suspension before ACK lysis (spleens only). Cells were stained for CD8 and CD11a and acquired on a FACSCalibur flow cytometer (BD Biosciences), and high expression of CD11a and low expression of CD8 were used to detect Ag-experienced cells as previously described (10). Surface marker expression among Ag-experienced (CD8^lo^/CD11a^hi^) and Ag-inexperienced (CD8^hi^/CD11a^lo^) CD8 T cells was determined by staining cells with CD8 and CD11a along with CD127 and KLRG1, CD62L, and CD27, or CD69. Cells were acquired on a FACSCanto flow cytometer (BD Biosciences) and analyzed using FlowJo software.

### CD8 and CD4 T Cell Depletion

For CD8 and CD4 T cell depletions, mice previously infected with Att LM (5 × 10^6^ CFU) were treated once with 800 µg of αCD8 (clone 2.43) or 400 µg of αCD4 (clone GK1.5) antibody (Ab) i.p. 5 days prior to Vir LM challenge. CD8 and CD4 T cell frequencies were assessed in peripheral blood lymphocytes (PBL) on day 0 prior to treatment and 2 days after treatment and found to be >99% depleted. Mice not receiving depleting Ab were given one matching dose of IgG Ab i.p. 5 days prior to challenge infection.

### Measure of Bacterial and Viral Clearance

For measure of bacterial clearance, LM-infected mice were sacrificed 4 or 5 days after challenge infection, and spleens and livers were harvested and placed in dH_2_0 containing 0.2% IGEPAL and disrupted using a tissue homogenizer. Samples were plated on tryptic soy broth (TSB)-agar plates containing streptomycin and incubated at 37°C for 24 h, and then CFUs were counted. As a measure of protection, body weight was monitored daily following challenge infections. For measure of viral clearance, blood was collected from LCMV-infected mice 4 days after infection, and serum was separated and collected by centrifugation of samples at 13,300 × *g* for 3 min. Additionally, spleens were harvested and placed in serum-free RPMI media and disrupted using a tissue homogenizer. 200 µL of supernatant was collected, and samples were flash frozen in liquid nitrogen. Viral titers were quantified with standard plaque assaying on VERO cells as previously described (13).

### Detection of Serum IFN-α, IFN-γ, and IL12

Blood was collected from mice at 24 and 72 h following LCMV infection and at 4, 24, and 48 h following Att LM infection, and serum was separated and collected by centrifugation of samples at 13,300 × *g* for 3 min. IFN-α was measured using a mouse IFN-α platinum ELISA kit and IL12 was measured using a mouse IL12 platinum ELISA kit (eBioscience). For detection of IFN-γ, purified IFN-γ mAb (eBioscience) was diluted to 2 μg/mL and 50 μL/well was added to a flat bottom 96 well MaxiSorp ELISA plate and incubated overnight at 4°C. The following day, the plate was washed with PBS/Tween, and 200 μL/well of RP10 was added and plates were incubated at room temperature for 2 h. Plates were then washed with PBS/Tween, and 25 µL of serum sample was added to wells along with 25 µL of PBS, and standards were prepared and plated with a range of 156.2–80,000 pg/mL, and plates were incubated overnight at 4°C. The following day, plates were washed with PBS/Tween. Biotinylated anti-IFN-γ detecting mAb (eBioscience) was diluted to 1 μg/mL in PBS, and 100 μL was added per well, and plates were incubated at room temperature for 2 h. Plates were then washed with PBS/Tween. Avidin-peroxidase was diluted to 2.5 µg/mL in PBS, and 100 µL was added per well and plates were incubated at room temperature for 30 min. Plates were then washed with PBS/Tween. 100 µL of TMB substrate containing 0.2 µL/mL of hydrogen peroxide was added per well and plates were incubated for 10 min at room temperature. The reaction was then stopped by adding 25 μL/well of 2 M sulfuric acid. Absorbance values (450 nM) were measured and assessed for all plates using Gen5 software (BioTek).

### Statistical Analysis

Statistical analyses were performed using GraphPad Prism software version 6 (GraphPad Software Inc., San Diego, CA, USA). Statistical comparisons of two groups were done using the unpaired *t*-test. Statistical comparisons of more than two groups were done using one-way ANOVA with the Bonferroni post-test. Statistical tests for variability of CD8 T cell responses of B6 compared to Swiss mice was done using an *F* test. *R*-squared values were calculated from linear regression analysis.

## Results

### Kinetics of Virus-Specific CD8 T Cell Responses in Inbred and Outbred Mice

**Figure 1:**
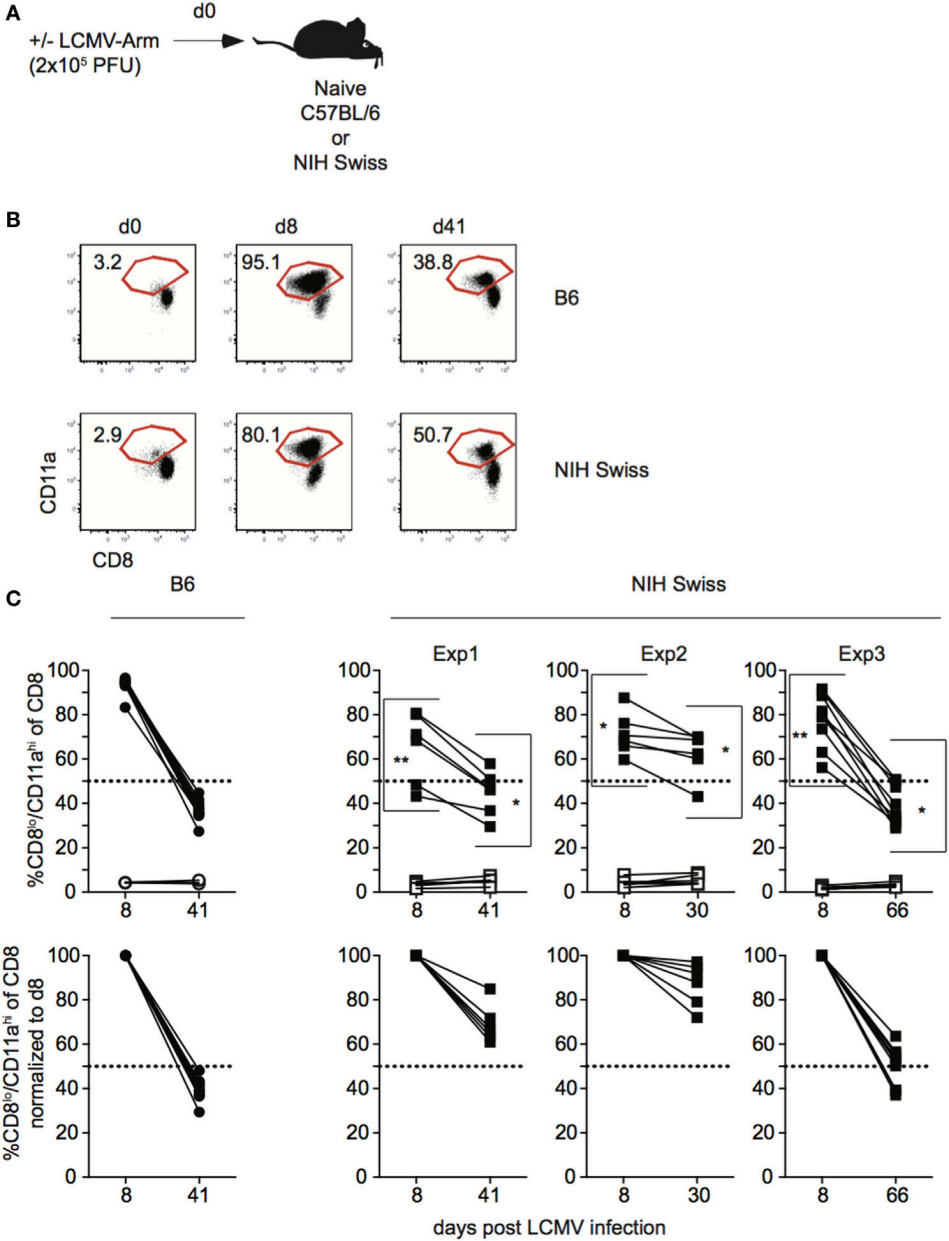
Magnitude of CD8 T cell responses following lymphocytic choriomeningitis virus (LCMV) infection is variable in individual outbred mice. **(A)** Experimental design. C57BL/6 (B6) or National Institutes of Health (NIH) Swiss mice were infected with 2 × 10^5^ plaque forming unit (PFU) LCMV-Armstrong. **(B)** Representative dot plots of detection of antigen-specific CD8 T cells in B6 or Swiss mice on the indicated day post infection based on CD8/CD11a staining. Numbers inside plots indicate the percentage of gated CD8 T cells that are CD8^lo^/CD11a^hi^. **(C)** Top-Percentage of CD8^lo^/CD11a^hi^ cells of gated CD8 T cells among peripheral blood lymphocytes for B6 or Swiss mice at the indicated day post infection. Open circles or squares are uninfected mice. Bottom: percentage of CD8^lo^/CD11a^hi^ cells of gated CD8 T cells normalized to day 8 (100%). Dotted line at 50%. *, statistically significant (*p* < 0.05); **, statistically significant (*p* < 0.01) differences in variation between B6 and Swiss mice as determined by *F* test. Representative data from greater than three independent experiments with at least five mice per group per experiment.

Data generated using inbred mouse strains have shown that CD8 T cell responses to various pathogens follow similar kinetics of expansion, contraction, and memory generation (15, 16). Recent work from our laboratory demonstrated that expression of CD8α and CD11a could be used to identify Ag-specific CD8 T cell responses in both inbred and outbred mouse strains (10). To further examine kinetics of polyclonal CD8 T cell responses, inbred C57B/6 (B6) and outbred NIH Swiss (Swiss) mice were infected with the Armstrong strain of LCMV (Figure 1A). LCMV elicits a robust CD8 T cell response, and at the peak of the response [day 8 as determined in B6 mice (17)], greater than 90% of all CD8 T cells among PBLs in B6 mice were responding to infection based on low expression of CD8α and high expression of CD11a (Figures 1B,C). Over the same time period, the percentage of CD8^lo^/CD11a^hi^ cells in uninfected mice was low and did not change (Figures 1B,C). This period of proliferative expansion was followed by contraction, the extent of which was similar in individual inbred mice, and at a memory time point, approximately 40% of CD8 T cells in inbred mice expressed an Ag-experienced phenotype and persisted as LCMV-specific memory CD8 T cells (Figures 1B,C). However, while CD8 T cells proliferated, contracted, and formed a memory CD8 T cell population following LCMV infection in all outbred Swiss mice, the percentages of Ag-experienced cells among CD8 T cells during the proliferative expansion phase, the extent of contraction, and size of the memory CD8 T cell pool were significantly more variable in individual outbred Swiss mice compared to inbred B6 mice (Figure 1C). Interestingly, responses for Swiss mice were normally distributed, reflecting a range of responses that might be expected in the diverse human population. The peak of CD8 T cell expansion to infection can vary in individual outbred hosts (10, 11) and snap-shot analysis obtained at a single time point [here at day 8 post infection (p.i.)] can underestimate the magnitude of the primary expansion, a notion of relevance in a situation when T cell responses are analyzed in cohorts of genetically diverse individuals.

Thus, CD8 T cell responses in general display the characteristics of expansion, contraction, and memory formation following LCMV infection in all analyzed mice. However, the kinetics of the CD8 responses to viral infection in individual outbred hosts differ significantly.

### Differences in Magnitude of CD8 T Cell Responses in Individual Swiss Mice Are Not Correlated with Inflammatory Cytokines Elicited or Pathogen Load

**Figure 2:**
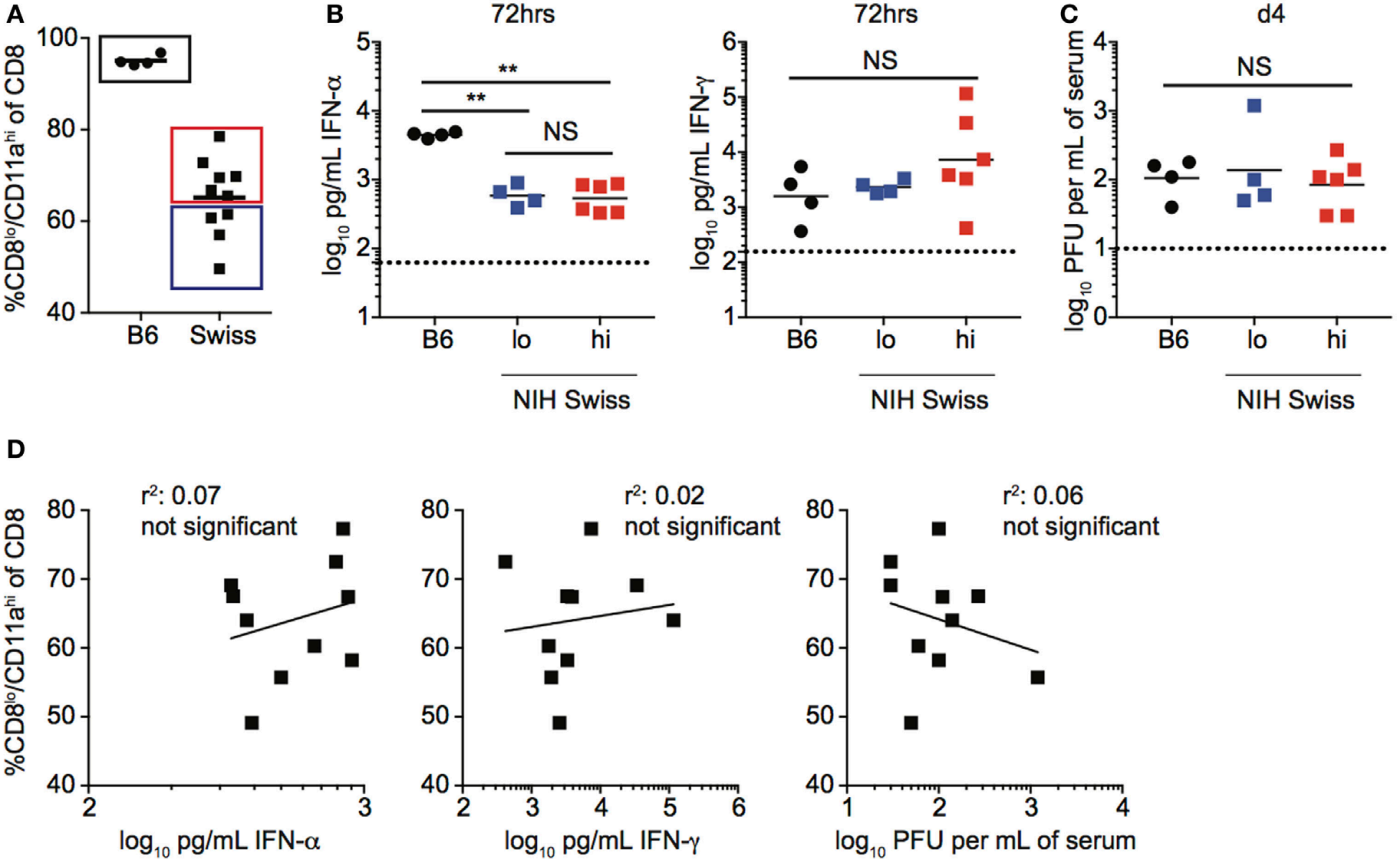
Magnitude of responses in Swiss mice is not correlated with inflammation or clearance of infection. **(A)** Percentage of CD8^lo^/CD11a^hi^ cells of gated CD8 T cells among peripheral blood lymphocytes (PBL) for B6 or Swiss mice at day 10 following lymphocytic choriomeningitis virus-Armstrong infection [2 × 10^5^ plaque forming unit (PFU)]. Swiss mice below the mean (lo) and above the mean (high) for percentage of CD8^lo^/CD11a^hi^ cells of gated CD8 T cells were split into two groups for panels **(B,C)**. **(B)** Concentration of IFN-α (left) or IFN-γ (right) detected in serum of infected B6 or Swiss mice at 72 h post infection. Dotted line indicates limit of detection. **(C)** Viral titers per mL of serum on day 4 following infection. Dotted line indicates limit of detection. **(D)** Percentage of CD8^lo^/CD11a^hi^ cells of gated CD8 T cells among PBL at day 10 following infection (*y* axis) relative to concentration of IFN-α (left) or IFN-γ (middle) detected in serum of infected B6 or Swiss mice at 72 h post infection, or viral titers per mL of serum on day 4 following infection (right) (*x* axis). NS, not statistically significant; **, statistically significant (*p* < 0.01) as determined by one-way ANOVA with a Bonferroni post-test. Statistical significance of *R*-squared values based on linear regression analysis. Representative data from one of two independent experiments with 4–10 mice per group.

The magnitude of the effector CD8 T cell response has been shown in inbred mice to be dependent upon the dose of infection as well as the amount of inflammation (18–22). To determine if infection is controlled similarly in inbred and outbred mice, and if the amount of inflammation elicited following infection could explain differences in the magnitude of CD8 T cell responses in individual Swiss mice, we infected B6 and Swiss mice with LCMV and examined viral titers and serum cytokine levels following infection (Figure S1A in Supplementary Material). While differences in amounts of IFN-α and IFN-γ present in serum and rate of viral clearance were seen in inbred compared to outbred mice (Figures S1B,C in Supplementary Material), infectious virus and inflammatory cytokines were detected in both types of mice, suggesting that all mice were infected. However, while the size of the effector CD8 T cell response, amount of inflammatory cytokines in serum, and rate of viral clearance were similar in individual inbred mice, they showed variability in individual outbred mice (Figure 2A; Figures S1B,C in Supplementary Material). To determine if the size of the effector CD8 T cell response in individual outbred mice was impacted by inflammation elicited in response to infection or viral load, we determined amounts of circulating cytokines and viral titers in serum for Swiss mice with high and low percentages of Ag-experienced CD8 T cells 10 days following infection. No statistically significant differences in serum concentrations of IFN-α and IFN-γ or viral titers in serum 4 days following infection were seen in outbred mice with high or low percentages of Ag-experienced effector cells (Figures 2B,C). Additionally, when we plotted the percentage of CD8^lo^/CD11a^hi^ cells in PBL 10 days following infection versus concentration of IFN-α or IFN-γ found in serum 3 days following infection or viral PFUs found in the serum 4 days following infection, we did not find a statistically significant correlation based upon linear regression analysis (Figure 2D). Taken together, these data suggest that differences in magnitude of effector CD8 T cell responses in individual outbred mice are not due to differences in viral load or amount of inflammation elicited in response to infection.

### Variability in Magnitude of CD8 T Cell Responses in Individual Swiss Mice Is Not Correlated with the Numbers of Naïve CD8 T Cells Available before Infection, Extends to Peripheral Tissues, and Is Not Normalized after Co-Housing

Beyond differences in inflammatory cytokines elicited and clearance of infection, factors including size of the naïve CD8 T cell pool, differences in commensal microflora, and representation of naïve and Ag-experienced cells prior to infection could account for variations in the magnitude of CD8 T cell responses in individual outbred mice. While variation in the size of the CD8 T cell pool in uninfected Swiss mice was observed, size of the CD8 T cell pool prior to infection did not correlate with magnitude of the response after infection (Figure S2A in Supplementary Material). Additionally, in the absence of deliberate exposure to microorganisms, CD8 T cells respond to commensal microbes leading to the generation of an Ag-experienced population in otherwise infection-naïve mice (23). While the percentage of CD8 T cells that display an Ag-experienced phenotype prior to infection is low (Figures 1B,C), differences in cells of an Ag-experienced phenotype prior to infection could limit the naïve cells available to respond to infection and lead to variation in magnitude of the CD8 T cell responses following infection. However, when we plotted the percentage of CD8^lo^/CD11a^hi^ or CD44^hi^ cells, another marker used to identify Ag-experienced CD8 T cells, in Swiss mice prior to infection, versus the percentage of CD8^lo^/CD11a^hi^ among CD8 T cells in PBL on day 8 following LCMV infection, we did not find a significant correlation (Figure S2B in Supplementary Material). This suggests that variations in the magnitude of CD8 T cell responses following infection in outbred mice are not due to differences in available naïve CD8 T cell pool prior to infection.

**Figure 3:**
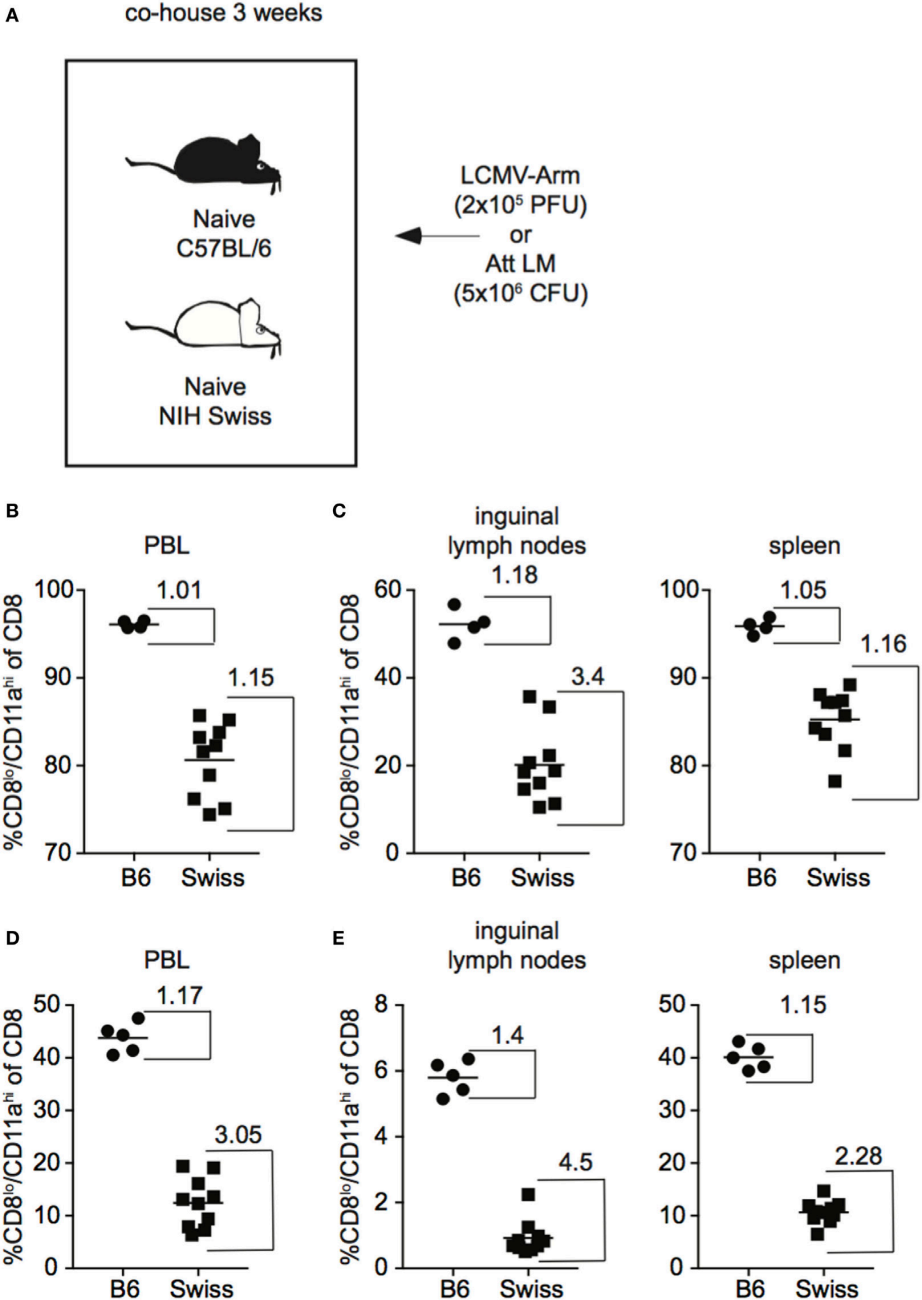
Magnitude of CD8 T cell responses following infection is variable in tissues of outbred mice and variation is not normalized following co-housing. **(A)** Experimental design. B6 or Swiss mice were co-housed for 3 weeks and infected with either 2 × 10^5^ plaque forming unit (PFU) lymphocytic choriomeningitis virus (LCMV)-Armstrong or 5 × 10^6^ colony forming unit (CFU) attenuated *actA*-deficient *Listeria monocytogenes* (Att LM). **(B)** Percentage of CD8^lo^/CD11a^hi^ cells of gated CD8 T cells among peripheral blood lymphocytes (PBL) for B6 or Swiss mice at day 8 after infection with LCMV-Armstrong. **(C)** Percentage of CD8^lo^/CD11a^hi^ cells of gated CD8 T cells among lymphocytes in inguinal lymph nodes (left) or among splenocytes (right) for B6 or Swiss mice at day 8 after infection with LCMV-Armstrong. **(D)** Percentage of CD8^lo^/CD11a^hi^ cells of gated CD8 T cells among PBL for B6 or Swiss mice at day 7 after Att LM infection. **(E)** Percentage of CD8^lo^/CD11a^hi^ cells of gated CD8 T cells among lymphocytes in inguinal lymph nodes (left) or among splenocytes (right) for B6 or Swiss mice at day 7 after Att LM infection. Numbers inside the graph represent the “inside group variability” that is calculated by dividing the highest with the lowest responders inside the group. Data from one experiment with 4–10 mice per group.

In addition to skewing the representation of naïve and Ag-experienced cells within the CD8 T cell pool, commensal microbes have been shown to impact the overall level of inflammation elicited upon infection (24), which could impact the size of the CD8 T cell response generated following infection. Because Swiss mice used in these studies originated at a different facility than B6 mice and not all Swiss mice in each experiment were littermates, it is possible that differences in commensal microflora were influencing CD8 T cell responses resulting in variation in the size of responses in individual Swiss mice. To test whether differences in commensal microflora might be impacting the CD8 T cell response in inbred and outbred mice, we co-housed B6 and Swiss mice for 3 weeks to normalize commensal microflora prior to infection with LCMV or Att LM (25) (Figure 3A). As seen in Figure 1, magnitude of effector CD8 T cell responses detected in PBL were more variable in Swiss compared to B6 mice even after co-housing (Figure 3B). Furthermore, greater variability of CD8 T cell responses was seen in individual Swiss compared to B6 mice in inguinal lymph nodes and spleens (Figure 3C), demonstrating that variability of responses in outbred mice extends to peripheral tissues. Variability of responses detected in PBL, inguinal lymph nodes, and spleens was also observed in individual Swiss mice following Att LM infection (Figures 3D,E). Taken together, these data suggest that variability in magnitudes of CD8 T cell responses to infection in outbred mice are not due to differences in availability of naïve CD8 T cells within the CD8 T cell pool or commensal microflora and extend to cells present in peripheral tissues.

### Development of Memory CD8 T Cells following Infection Does Not Occur at a Predictable Rate in Outbred Mice

**Figure 4:**
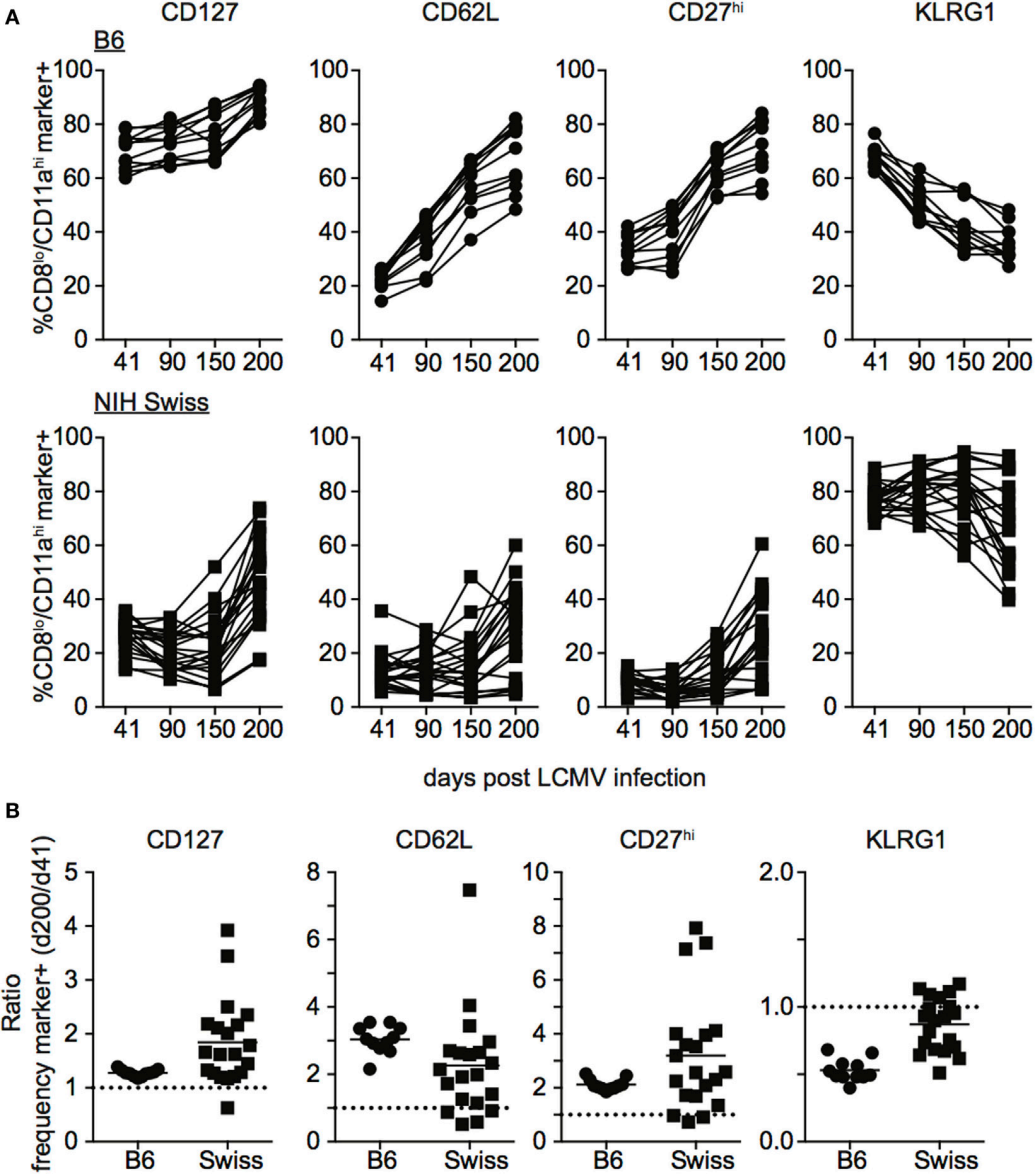
Rate of phenotypic progression following lymphocytic choriomeningitis virus (LCMV) infection is variable in individual outbred mice. **(A)** Percentage of gated CD8^lo^/CD11a^hi^ cells expressing the indicated marker for individual B6 (top) or Swiss (bottom) mice on the indicated day after LCMV infection. **(B)** Ratio of the percentage of CD8^lo^/CD11a^hi^ CD8 T cells expressing the indicated surface protein at day 200 over the percentage of CD8^lo^/CD11a^hi^ CD8 T cells expressing the indicated surface protein at day 41 for B6 and Swiss mice. Dotted line at 1. Representative data from greater than three independent experiments with at least five mice per group per experiment.

During the effector differentiation process, CD8 T cells modulate expression of surface proteins including CD127, CD62L, CD27, and KLRG1. Expression of these markers has been correlated with CD8 T cell function (26–31), and we and others have shown that the phenotype and function of memory CD8 T cells evolves with time following infection (32–36). To determine how the phenotype of memory CD8 T cells generated following infection compares between inbred and outbred mice, we examined expression of CD127, CD62L, CD27, and KLRG1 among CD8^hi^/CD11a^lo^ Ag-inexperienced and CD8^lo^/CD11a^hi^ Ag-experienced CD8 T cells at various times following LCMV infection. As expected, CD8^hi^/CD11a^lo^ cells in both inbred and outbred mice displayed the phenotype of naïve CD8 T cells, including expression of CD127, CD62L, and CD27 and lack of expression of KLRG1 at all time points examined (Figures S3A,B in Supplementary Material). In addition, expression of CD127, CD62L, and CD27 progressively increased and expression of KLRG1 progressively decreased with time after infection among CD8^lo^/CD11a^hi^ cells in individual inbred mice (Figure 4A). Interestingly, expression of CD127, CD62L, CD27, and KLRG1 among Ag-experienced CD8 T cells was different in individual outbred mice, and these differences were magnified as time passed after infection (Figure 4A). While the ratio for CD127, CD62L, and CD27 was greater than 1 (i.e., expression increased with time when expression at day 200 post infection was compared to expression at day 41) and the ratio for KLRG1 was less than 1 (i.e., expression decreased with time) in each individual inbred mouse and most outbred mice, the ratio for CD127, CD62L, and CD27 was near or below 1 and the ratio for KLRG1 was near or above 1 in some outbred mice (Figure 4B), suggesting that the phenotype of memory CD8 T cells does not progress or progresses very slowly with time after infection in some outbred mice.

Phenotypic progression or non-progression of memory CD8 T cells in outbred hosts appeared to be correlated to the size of the memory CD8 T cell pool generated following infection, as mice with high levels of CD127, CD62L, and CD27 and low levels of KLRG1 at day 121 following infection had lower levels of memory CD8 T cells than mice with low expression of CD127, CD62L, and CD27 and high expression of KLRG1 (Figures S4A,B in Supplementary Material). There were no overt signs of re-infection of mice during the studies, and while we cannot rule out infection at distal sites or responses to endogenous microbes, periodic staining for CD69 demonstrated that Ag-experienced CD8 T cells did not express CD69, a marker of recent activation (Figure S4C in Supplementary Material), suggesting that systemic infection was not occurring and that LM infection is not persistent in outbred mice. Taken together, these data show that while the phenotypic progression of memory CD8 T cells following infection in some outbred mice occurs in a similar fashion to inbred mice, progression occurs at different rates in individual outbred mice.

### Kinetics of Bacteria-Specific CD8 T Cell Responses in Inbred and Outbred Mice

**Figure 5:**
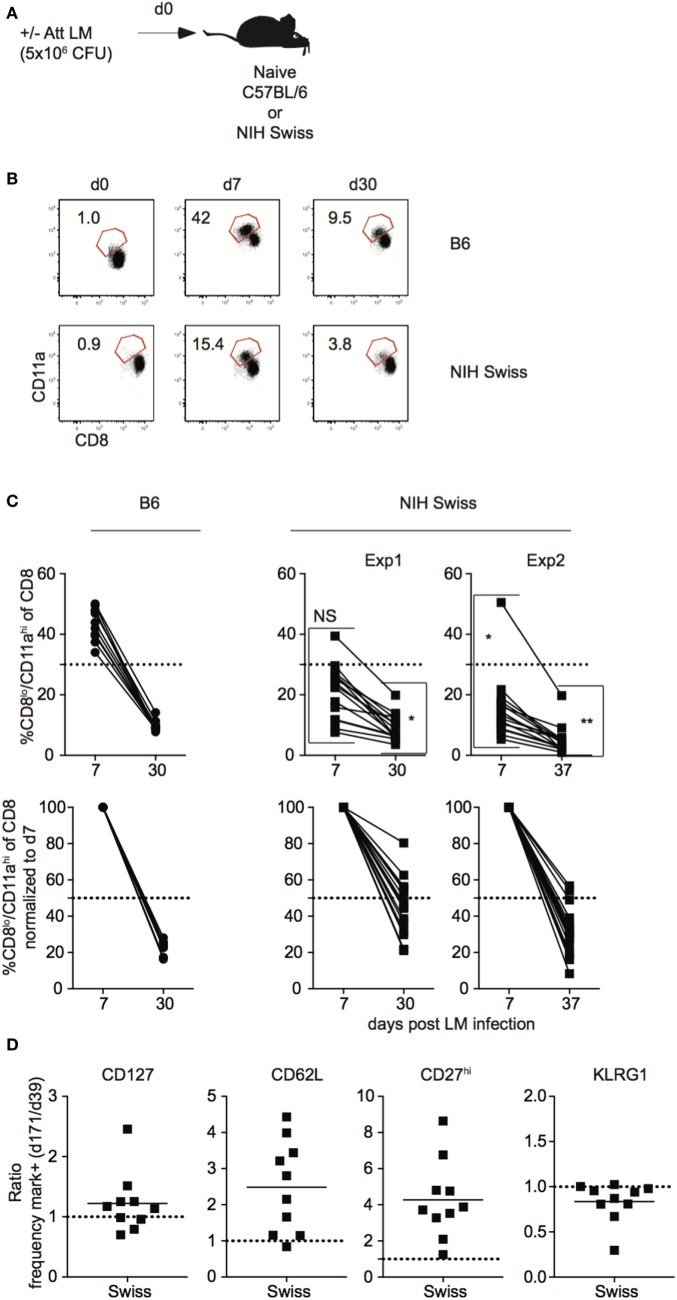
Magnitude of CD8 T cell responses following LM infection is variable in individual outbred mice. **(A)** Experimental design. B6 or Swiss mice were infected with 5 × 10^6^ colony forming unit (CFU) attenuated *actA*-deficient *Listeria monocytogenes* (Att LM). **(B)** Representative dot plots of detection of antigen-specific CD8 T cells in B6 or Swiss mice on the indicated day post infection based on CD8/CD11a staining. Numbers inside plots indicate the percentage of gated CD8 T cells that are CD8^lo^/CD11a^hi^. **(C)** Top: percentage of CD8^lo^/CD11a^hi^ cells of gated CD8 T cells among peripheral blood lymphocytes (PBL) for B6 or Swiss mice at the indicated day post infection. Bottom: percentage of CD8^lo^/CD11a^hi^ cells of gated CD8 T cells normalized to day 7 (100%). Dotted line at 50%. **(D)** Ratio of the percentage of CD8^lo^/CD11a^hi^ CD8 T cells expressing the indicated surface protein at day 171 over the percentage of CD8^lo^/CD11a^hi^ CD8 T cells expressing the indicated surface protein at day 39 for Swiss mice. Dotted line at 1. NS, not statistically significant; *, statistically significant (*p* < 0.05); **, statistically significant (*p* < 0.01) differences in variation between B6 and Swiss mice as determined by *F* test. Representative data from greater than three independent experiments with at least five mice per group per experiment.

In order to generalize and extend findings obtained in outbred hosts after LCMV infection, a similar set of experiments was performed in inbred and outbred mice following *L. monocytogenes* infection. Naïve B6 and Swiss mice were challenged with Att LM and kinetics of Ag-experienced CD8 T cells was analyzed (Figure 5A). While the size of the effector and memory responses were lower following LM infection compared to LCMV infection (Figures 1B,C and 5B,C), similar to LCMV infection, the extent of proliferative expansion, degree of contraction, and size of the resulting memory CD8 T cell pool following LM infection were similar in individual inbred mice but significantly more variable in individual outbred mice (Figures 5B,C). Again, differences in amount of inflammatory cytokines (IFN-γ and IL12) elicited in response to LM infection and pathogen load were seen between inbred and outbred mice (Figure S5 in Supplementary Material), but differences in magnitude of effector CD8 T cell responses in individual outbred mice appeared unlikely to be caused by differences in infection or amount of inflammation elicited in response to infection in individual outbred mice. Importantly, similar to LCMV infection, the rate of phenotypic progression of memory CD8 T cells following infection differed in individual outbred mice (Figure 5D). Thus, size of the effector and memory pool and phenotype of memory CD8 T cells that develop in response to LM infection is variable in individual outbred mice.

### CD8 T Cells Contribute to Immune-Mediated Protection against LM in Inbred and Outbred Mice

**Figure 6:**
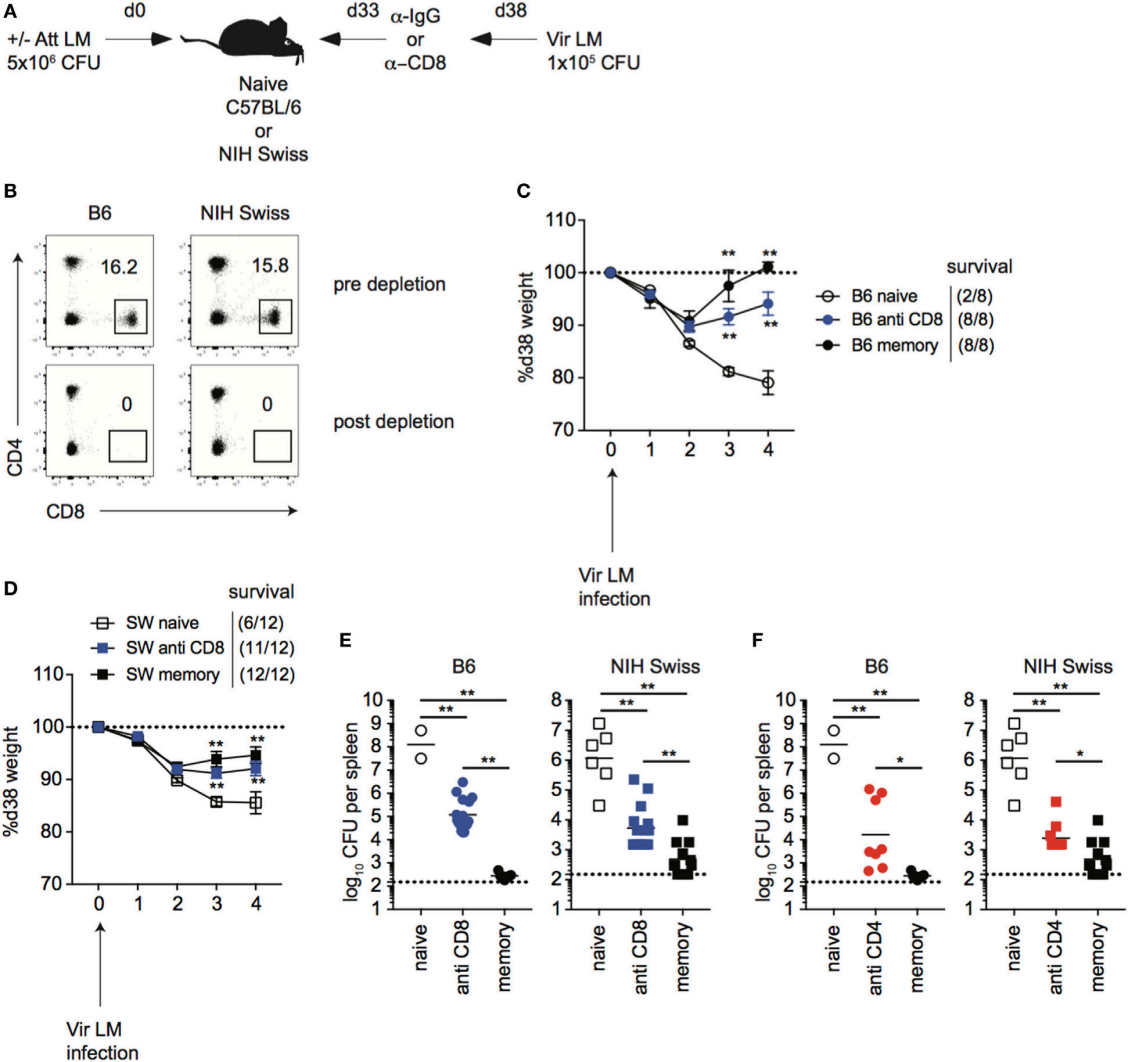
CD8 T cells contribute to immune-mediated protection against LM in inbred and outbred mice. **(A)**. Experimental design. B6 or Swiss mice were not infected (naïve), or given 1° infection of 5 × 10^6^ colony forming unit (CFU) attenuated *actA*-deficient *Listeria monocytogenes* (Att LM) (memory). 33 days later, mice were given injection of IgG (naïve and memory) or αCD8 Abs (memory—800 μg/mouse i.p.). Mice were challenged with 1 × 10^5^ CFU virulent *Listeria monocytogene* (Vir LM) 38 days following 1° infection. **(B)** Representative dot plots of CD4 and CD8 T cells among peripheral blood lymphocytes (PBL) for B6 or Swiss mice prior to CD8 T cell depletion (top) and 2 days post αCD8 injection (bottom). **(C)** Weight of B6 mice that received the indicated 1° infection and antibody (Ab) treatment on the indicated day after challenge infection as a percentage of weight prior to challenge infection. Dotted line at 100% (weight prior to challenge infection). Survival within groups is indicated in the figure legend. **(D)** Weight of Swiss mice that received the indicated 1° infection and Ab treatment on the indicated day after challenge infection as a percentage of weight prior to challenge infection. Dotted line at 100% (weight prior to challenge infection). Survival within groups is indicated in the figure legend. **(E)** Bacterial titers per spleen for B6 (left) or Swiss (right) mice that received the indicated 1° infection and Ab treatment 4 days after challenge infection. Dotted line indicates limit of detection. **(F)** B6 or Swiss mice were treated as depicted in panel **(A)**, but were given αCD4 Abs (memory—400 μg/mouse i.p.). Bacterial titers per spleen for B6 (left) or Swiss (right) mice that received the indicated 1° infection and Ab treatment 4 days after challenge infection. Dotted line indicates limit of detection. **, statistically significant (*p* < 0.01) as determined by one-way ANOVA with a Bonferroni post-test. Error bars indicate SEM. Data from one experiment with eight to 12 mice per group.

While immune-mediated protection against LM has been shown to be mediated by CD8 T cells in inbred mice (37–40), it is unknown if this is the case for outbred mice. To determine this, naïve B6 and Swiss mice were either infected or not infected with Att LM, and, at a memory time point, mice were treated with either control IgG or with anti-CD8 mAb before secondary challenge with virulent LM (Figures 6A,B). Both inbred and outbred groups of immunized mice that were depleted of CD8 T cells lost less weight (Figures 6C,D) and had significantly less CFUs in spleen and liver 4 days following re-challenge (Figure 6E) than non-immunized naïve mice, suggesting that cells other than memory CD8 T cells contribute to immune-mediated protection against LM. However, with this depletion strategy, we could not rule out that tissue-resident memory CD8 T cells, which are harder to deplete than circulating cells (41), accounted for the difference in protection between immunized mice that were depleted of CD8 T cells and non-immunized mice. To provide further evidence that cells other than memory CD8 T cells contribute to memory-mediated protection against LM infection, we depleted CD4 T cells from mice that were previously infected with Att LM prior to challenge. As with CD8 T cell depletion, mice that were depleted of CD4 T cells had lower recoverable bacteria from spleens than non-immunized mice, but more than mice that were immunized but not depleted of CD4 T cells, suggesting that CD4 T cells also contribute to memory-mediated protection against LM (Figure 6F). Importantly, however, CFUs in spleens and livers of mice previously infected with Att LM that were not depleted of CD8 T cells were significantly lower than mice depleted of CD8 T cells (Figure 6E), indicating that memory CD8 T cells significantly contribute to immune-mediated protection against LM in inbred as well in outbred mice.

### Size of the Memory Pool Generated Correlates with Degree of Protection against Re-Infection in Inbred Mice, But Not All Outbred Mice

**Figure 7:**
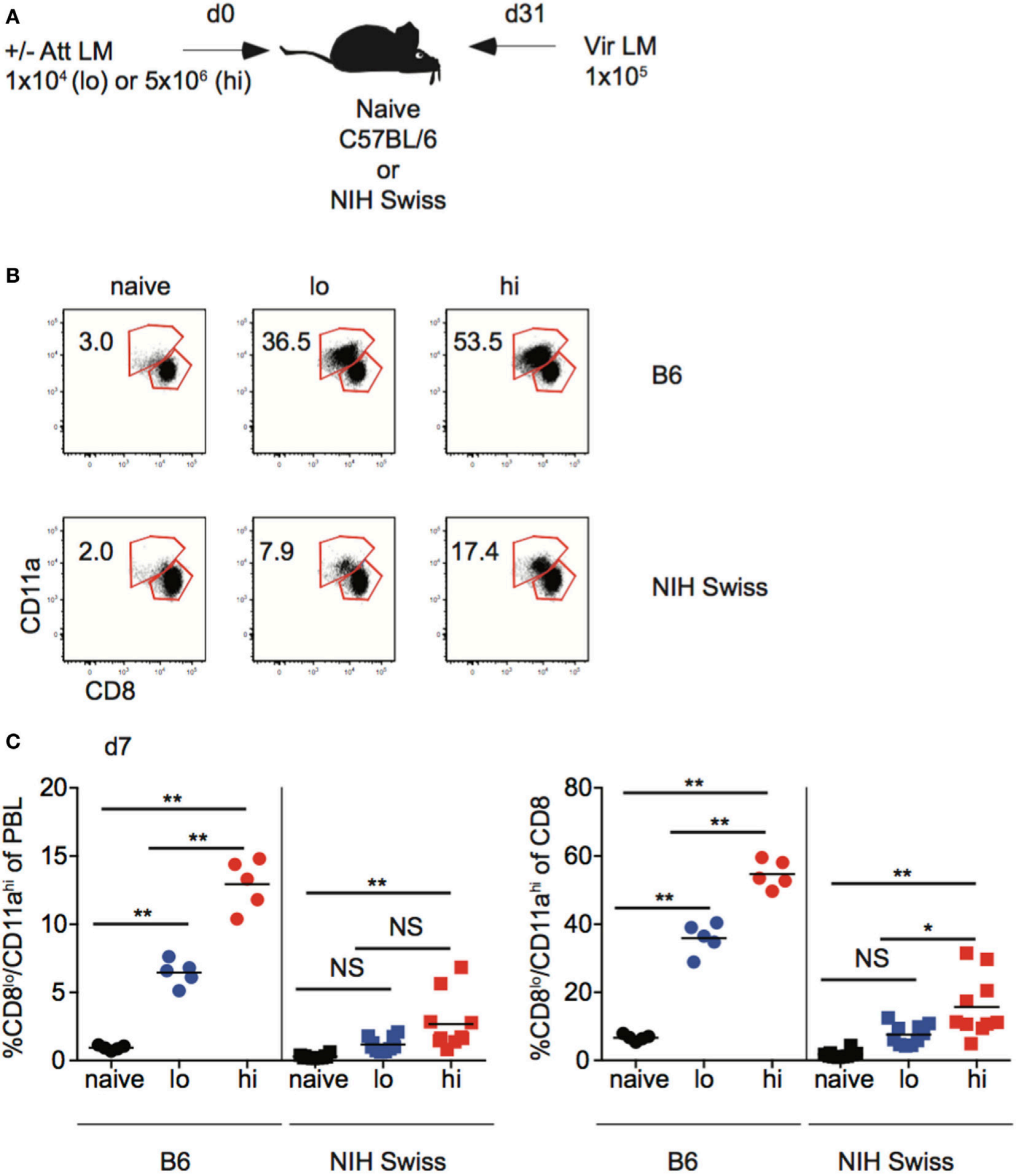
Effector pool size in outbred mice does not always correlate with infection dose. **(A)** Experimental design. B6 or Swiss mice were not infected (naïve), or infected with 1 × 10^4^ colony forming unit (CFU) (lo) or 5 × 10^6^ CFU (hi) attenuated *actA*-deficient *Listeria monocytogenes* (Att LM). 31 days following 1° infection mice were challenged with 1 × 10^5^ CFU virulent *Listeria monocytogene* (Vir LM). **(B)** Representative dot plots of detection of antigen-specific CD8 T cells in B6 or Swiss mice that received the indicated 1° infection dose on day 7 after infection based upon CD8/CD11a staining. Numbers inside plots indicate the percentage of gated CD8 T cells that are CD8^lo^/CD11a^hi^. **(C)** Left: percentage of CD8^lo^/CD11a^hi^ cells of peripheral blood lymphocytes (PBL) for B6 or Swiss mice that received the indicated 1° infection dose at day 7 after infection. Right: percentage of CD8^lo^/CD11a^hi^ cells of gated CD8 T cells among PBL for B6 or Swiss mice that received the indicated 1° infection dose at day 7 after infection. NS, not statistically significant; *, statistically significant (*p* < 0.05); **, statistically significant (*p* < 0.01) as determined by one-way ANOVA with a Bonferroni post-test. Representative data from one of three independent experiments with 5–10 mice per group.

Studies conducted using inbred mice have shown that the magnitude of effector CD8 T cell responses and size of the memory CD8 T cell pool generated is related to the dose of infection and/or vaccination (22). Also, the degree of CD8 T cell-mediated protection is dependent upon the number of memory CD8 T cells present at the time of re-infection (42, 43). To examine if those relationships that are critical for design and implementation of effective vaccines hold true in diverse recipients, naïve B6 and Swiss mice were infected with a low dose (1 × 10^4^ CFU) or a high dose (5 × 10^6^ CFU) of Att LM, and at a memory time point 31 days later mice were challenged with Vir LM (Figure 7A). An additional group of naive mice served as non-immune controls.

Upon infection with Att LM, the magnitude of the effector CD8 T cell response in inbred mice increased with increasing infection dose (Figures 7B,C). However, while effector responses of some outbred mice given high dose Att LM infection were higher than those that received low dose infection, this was not the case for all outbred mice (Figure 7C), suggesting that the magnitude of effector CD8 T cell responses does not always correlate with infection dose in outbred mice.

**Figure 8:**
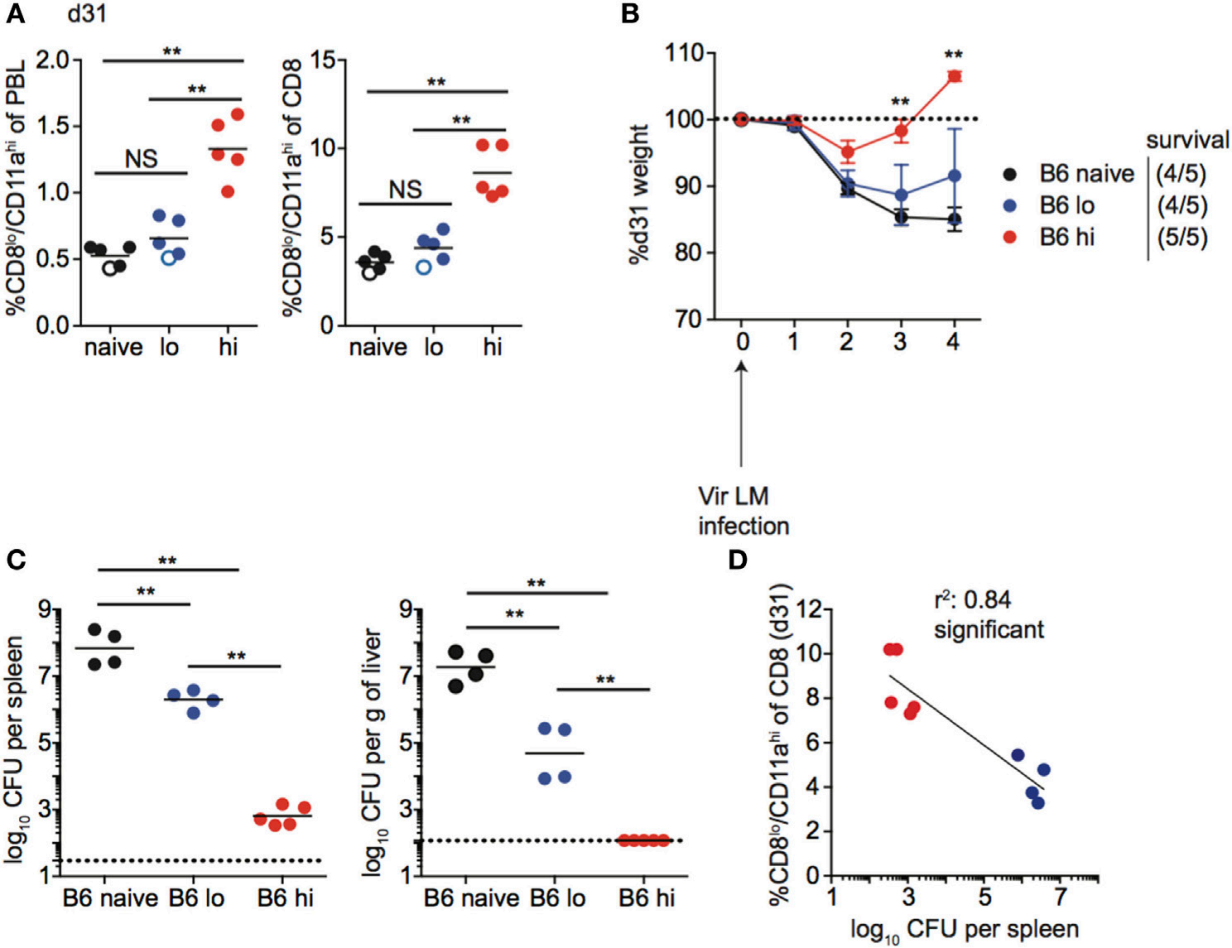
Memory CD8 T cell pool size correlates with protection in inbred mice. **(A)** Left: percentage of CD8^lo^/CD11a^hi^ cells of peripheral blood lymphocytes (PBL) for B6 mice that received the indicated 1° infection dose at day 31 after infection. Right: percentage of CD8^lo^/CD11a^hi^ cells of gated CD8 T cells among PBL for B6 mice that received the indicated 1° infection dose at day 31 after infection. Open circles indicate mice that died on or before day 4 following challenge infection. **(B)** Weight of B6 mice that received the indicated 1° infection dose on the indicated day after challenge infection as a percentage of weight prior to challenge infection. Dotted line at 100% (weight prior to challenge infection). Survival within groups is indicated in the figure legend. **(C)** Bacterial titers per spleen (left) or per g of liver (right) for B6 mice that received the indicated 1° infection dose on day 4 following challenge infection. Dotted line indicates limit of detection. **(D)** Percentage of CD8^lo^/CD11a^hi^ cells of gated CD8 T cells among PBL prior to challenge infection (*y* axis) relative to bacterial titers recovered in spleens of mice day 4 following challenge infection (*x* axis) for B6 mice that received low dose 1° infection (blue circles) and high dose 1° infection (red circles). NS, not statistically significant; **, statistically significant (*p* < 0.01) as determined by one-way ANOVA with a Bonferroni post-test. Statistical significance of *R*-squared values based on linear regression analysis. Error bars represent SEM. Representative data from one of three independent experiments with five mice per group.

Following contraction, the size of the memory CD8 T cell pool generated was related to infection dose in inbred mice, as mice that received high-dose Att LM infection had a higher percentage of Ag-experienced CD8 T cells among PBL and among CD8 T cells at a memory time point (Figure 8A). Importantly, upon re-challenge the degree of protection observed correlated with the initial dose of Att LM infection. While a similar percentage of naïve and mice that received low-dose LM infection survived Vir LM infection, all mice that received high-dose Att LM infection survived the challenge infection (Figures 8A,B). However, even mice vaccinated with low dose of Att LM had 90–99% less bacteria in spleen and liver compared to non-immunized controls (Figure 8C). When we interrogated whether the extent of protection as measured by bacteria recovered from the spleen 4 days after challenge was dependent upon the size of the LM-specific memory CD8 T pool prior to challenge infection, a statistically significant correlation was observed (Figure 8D).

Taken together, these data suggest that the magnitude of the effector response and size of the memory CD8 T cell pool achieved following infection (vaccination) is dependent on infection dose in inbred mice, and that the degree of memory CD8 T cell-mediated protection correlates with the size of the memory CD8 T cell pool prior to challenge.

**Figure 9:**
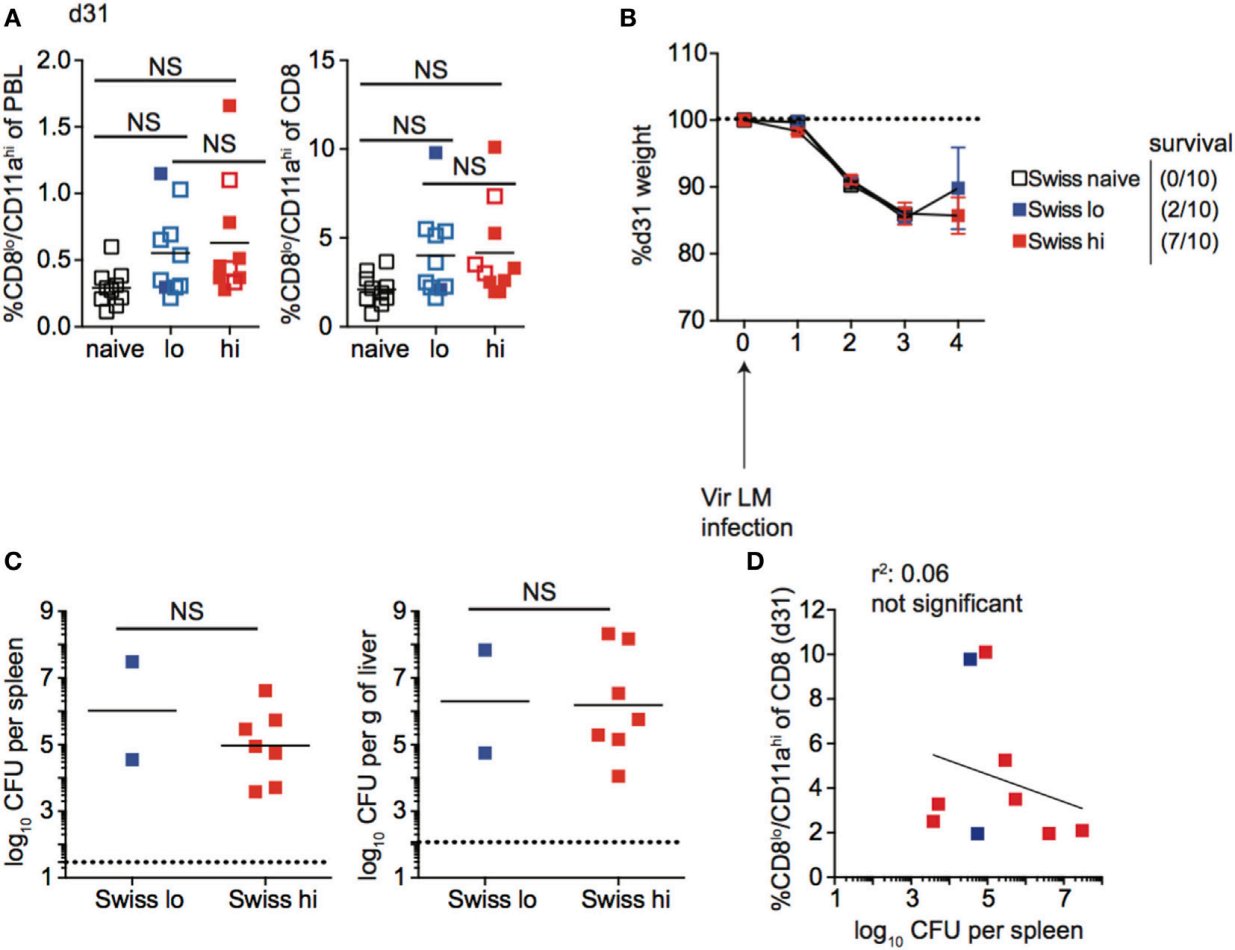
Memory CD8 T cell pool size does not correlate with protection in all outbred mice. **(A)** Left: percentage of CD8^lo^/CD11a^hi^ cells of peripheral blood lymphocytes (PBL) for Swiss mice that received the indicated 1° infection dose at day 31 after infection. Right: percentage of CD8^lo^/CD11a^hi^ cells of gated CD8 T cells among PBL for Swiss mice that received the indicated 1° infection dose at day 31 after infection. Open circles indicate mice that died on or before day 4 following challenge infection. **(B)** Weight of Swiss mice that received the indicated 1° infection dose on the indicated day after challenge infection as a percentage of weight prior to challenge infection. Dotted line at 100% (weight prior to challenge infection). Survival within groups is indicated in the figure legend. **(C)** Bacterial titers per spleen (left) or per g of liver (right) for Swiss mice that received the indicated 1^o^ infection dose on day 4 following challenge infection. Only two Swiss mice that received low dose attenuated *actA*-deficient *Listeria monocytogenes* (Att LM) infection survived to day 4 post challenge infection to determine bacterial titers. Dotted line indicates limit of detection. **(D)** Percentage of CD8^lo^/CD11a^hi^ cells of gated CD8 T cells among PBL prior to challenge infection (*y* axis) relative to bacterial titers recovered in spleens of mice at day 4 following challenge infection (*x* axis) for Swiss mice that received low-dose 1° infection (blue squares) and high dose 1° infection (red squares). NS, not statistically significant as determined by Student’s *t*-test or one-way ANOVA with a Bonferroni post-test. Statistical significance of *R*-squared values based on linear regression analysis. Error bars represent SEM. Representative data from one of three independent experiments with 10 mice per group.

Unlike inbred mice, a relationship between the magnitude of the effector CD8 T cell response and the dose of Att LM infection was not observed for all outbred mice (Figure 7C). Similarly, the size of the memory CD8 T cell pool did not correspond with infection dose in all outbred mice, as the size of the memory CD8 T cell pool for some mice that received low dose Att LM infection was similar to or even greater than that of mice that received high-dose Att LM infection (Figure 9A). While decreased susceptibility to LM infection in outbred compared to inbred mice could account for reduced memory CD8 T cell responses following Att LM infection, lower bacterial titers in Swiss compared to B6 mice were not seen in all experiments (Figures 6, 8 and 9; Figure S5 in Supplementary Material), and likely point to differences in cohorts of outbred Swiss mice. Previous infection with Att LM provided a survival advantage following challenge that was dependent on infection dose, as while all LM-naïve mice died following challenge infection, at least some mice from both groups that received previous Att LM infection lived, and a greater percentage of mice that received high-dose Att LM infection survived compared to mice that received low-dose Att LM infection (Figures 9A,B). However, mice that received high or low dose Att LM infection lost a similar amount of weight following challenge infection, and similar CFUs of bacteria were recovered from surviving mice of both groups after challenge infection (Figures 9B,C). When the percentage of Ag-experienced CD8 T cells prior to challenge infection was plotted against bacteria recovered from the spleens of mice four days after challenge, there was no statistically significant correlation between the size of the memory CD8 T pool prior to challenge and clearance of bacteria (Figure 9D). Furthermore, in the particular experiment when we examined outbred mice that survived challenge infection, we noted that one of the surviving mice from the low-dose Att LM infection group had among the smallest sized memory CD8 T cell pool prior to challenge infection (Figure 9A). Additionally, one mouse from the high-dose Att LM infection group that died following challenge infection had among the highest sized memory pool following Att LM infection (Figure 9A). Both of these observations support the conclusion that size of the memory CD8 T cell pool prior to challenge does not always correlate with degree of protection in all outbred mice.

### Booster Infection to Increase the Memory CD8 T Cell Pool Size Provides All Outbred Mice with Increased Protection against Re-Infection

**Figure 10:**
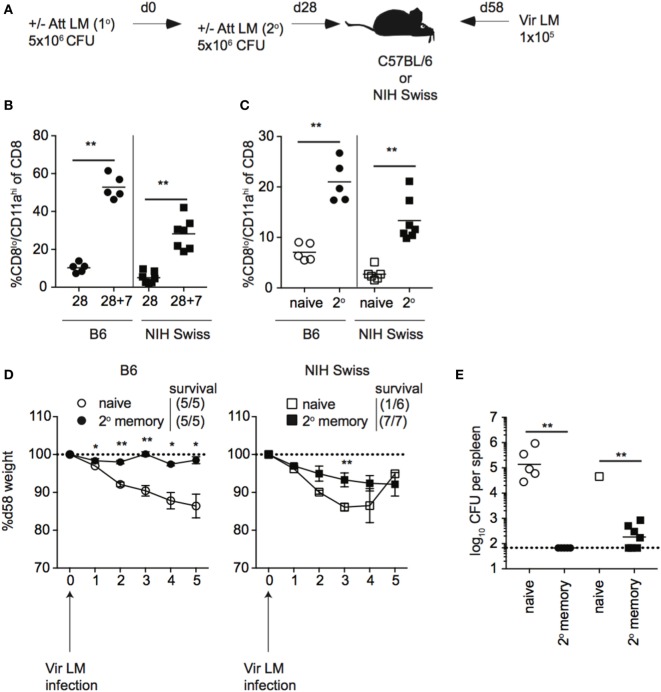
2° booster infection increases the size of the memory CD8 T cell pool and provides protection to all outbred mice. **(A)** Experimental design. B6 or Swiss mice were not infected (naïve) or infected with 5 × 10^6^ colony forming unit (CFU) attenuated *actA*-deficient *Listeria monocytogenes* (Att LM). 28 days later, mice received a 2° infection with 5 × 10^6^ CFU Att LM. 30 days following the 2° infection, mice were challenged with 1 × 10^5^ CFU virulent *Listeria monocytogene* (Vir LM). **(B)** Percentage of CD8^lo^/CD11a^hi^ cells of gated CD8 T cells among peripheral blood lymphocytes (PBL) for B6 or Swiss mice at day 28 following 1° infection and 7 days following 2° infection (28 + 7). **(C)** Percentage of CD8^lo^/CD11a^hi^ cells of gated CD8 T cells among PBL for naïve B6 or Swiss mice and at day 30 following 2° infection. **(D)** Weight of B6 (left) and Swiss (right) mice that received either no infection or 2° Att LM infection on the indicated day after challenge infection as a percentage of weight prior to challenge infection. Dotted line at 100% (weight prior to challenge infection). Survival within groups is indicated in the figure legend. **(E)** Bacterial titers per spleen for B6 and Swiss mice that received either no infection or 2° Att LM infection on day 5 following challenge infection. Dotted line indicates limit of detection. *, statistically significant (*p* < 0.05); **, statistically significant (*p* < 0.01) as determined by Student *t*-test or one-way ANOVA with a Bonferroni post-test. Representative data from one of two independent experiments with five to seven mice per group.

Unlike B6 mice, we did not see a correlation between the size of the memory CD8 T cell pool generated following a single Att LM infection prior to challenge and protection against re-infection in outbred mice. However, the size of the memory CD8 T cell pool generated following Att LM infection was smaller in Swiss mice than B6 mice (Figures 8A and 9A), and not significantly different between groups of naïve Swiss mice and Swiss mice that received Att LM infection (Figure 9A). This caused us to question whether generating a memory CD8 T cell population of sufficient size could provide a consistent level of protection in outbred mice. To examine this, we infected naïve B6 and Swiss mice with Att LM, rested mice for approximately 1 month before infecting a second time with Att LM, and challenged mice with Vir LM infection 1 month after 2^o^ LM infection (Figure 10A). 2° Att LM infection resulted in a 2° CD8 T cell response as percentages of CD8^lo^/CD11a^hi^ cells were increased 7 days following 2° Att LM infection in both inbred and outbred mice (Figure 10B), and while size of the memory CD8 T cell pool was still variable in individual Swiss mice, resulted in a larger memory CD8 T cell pool that was significantly different between naïve Swiss mice and Swiss mice that received 2° Att LM infection (Figure 10C). Both inbred and outbred mice that received 2° Att LM infection lost less weight than naïve mice (Figure 10D), and unlike 1^o^ Att LM infection (Figure 9B), all Swiss mice were protected from infection-induced mortality. Furthermore, both inbred and outbred mice that received 2° Att LM infection had lower recoverable bacterial titers in spleens 5 days after challenge infection than mice that did not receive Att LM infection (Figure 10E). Therefore, while protection against re-infection of outbred mice does not always correlate with size of the memory CD8 T cell pool, it is possible to generate a large enough memory CD8 T cell pool in outbred mice to provide a consistent level of protection against re-infection.

## Discussion

Experiments in mice have proven invaluable for study of the immune system and have led to the development of clinical therapies for treatment of cancer and autoimmunity, and the development of vaccines to prevent the spread of infectious diseases. However, differences between mouse models and the normal human condition, including environmental exposure to infection and genetic diversity, both of which are limited in the majority of mouse immunology studies but abundant in the human population, may lead to an inaccurate representation of the immune system in health and disease states. Recent work has suggested that, including environmental exposure to pathogens or deliberate infection of inbred mice with microbes that humans are commonly exposed to could more accurately reflect the human immune system and could increase the translational potential of mouse models (7, 8). While inbred mice are extremely valuable for immunologic studies due to the tools that have been developed allowing for detailed analysis of the immune response, our current study suggests that inclusion of outbred mice in immunological studies may provide a more accurate representation of the immune response that occurs in the human population and should be used to complement studies conducted in inbred mice. Additionally, this study further advances the use of the surrogate activation approach to track CD8 T cell responses in any mouse strain and suggests that it may be suitable to investigate underlying causes of diversity in immune outcomes in genetically diverse mouse strains, such as those within the collaborative cross (44, 45).

The design of vaccines that stimulate protective memory CD8 T cells has the potential to reduce healthcare burdens caused by infectious disease, and our current study has important implications for the design and assessment of vaccines intended to illicit protective immunity following vaccination in outbred populations. Through work using inbred mouse strains, it has been recognized that memory CD8 T cell-mediated protection against re-infection depends on numbers, quality or functional abilities, and location of the memory CD8 T cells present following infection (46–49). Therefore, research has focused on generating memory CD8 T cells of sufficient quality and quantity through vaccination to provide protection against re-infection with pathogens of interest. In this study, we found that a primary infection strategy that led to the generation of a uniformly sized memory CD8 T cell pool of a similar phenotype and to increased protection against re-infection in all inbred mice led to the generation of memory pools of disparate sizes and phenotypes in individual outbred mice and did not protect all outbred mice following re-infection. This suggests that the success of vaccines described in inbred mice and the correlates of CD8 T cell-mediated protection based on the vaccine tested are likely more complex when examined in outbred populations. Future studies utilizing outbred mouse strains may inform the correlates of CD8 T cell-mediated protection following vaccination of genetically diverse populations.

We found that the size of the effector and memory CD8 T cell pool following infection, as well as the degree of immune-mediated protection, is variable in individual outbred mice, and the question remains as to why this occurs. Evidence suggests that the size of effector CD8 T cell responses generated following infection is dictated by the number of naïve CD8 T cell precursors recruited into the response (50, 51). Therefore, a possible explanation for differences in the magnitude of CD8 T cell responses and size of the memory CD8 T cell pool generated in individual mice is differences in precursor frequency of CD8 T cells recognizing Ags expressed by the invading pathogen, or differences in recruitment of naïve CD8 T cells into the effector response. Furthermore, studies have convincingly shown the important role that inflammation plays in eliciting robust CD8 T cell responses (18, 19, 52, 53). However, while differences in the amount of inflammatory cytokines elicited following infection were seen in individual outbred mice, we were unable to find a correlation between the amount of inflammatory cytokines elicited following infection and the size of the effector CD8 T cell pool in outbred mice.

It has also become increasingly apparent that commensal microflora has a large influence on CD8 T cell responses. Microflora has been shown to influence the naïve CD8 T cell repertoire (23), and while our data did not show a correlation between the size of the Ag-experienced compartment prior to infection and size of the CD8 T cell response following infection, further exploration of how commensal flora impacts naïve CD8 T cell repertoire and how this might influence the CD8 T cell response to infection in outbred mice is warranted. Additionally, while our co-housing experiments showed that variability in the size of the CD8 T cell response to infection was observable even when mice were co-housed to normalize microflora, it will be important to study if individual outbred mice show diversity in their microbiome and how this might impact the immune response of unique outbred mice.

It is also possible that differences in the genetic composition of individual outbred mice might contribute to differences in effector and memory CD8 T cell pool sizes generated following infection. Future studies should be conducted to identify genetic factors that may contribute to determining the size of the CD8 T cell response following infection. The use of collaborative cross mice, a group of mouse strains of distinct but known genetics that were engineered through interbreeding of five inbred mouse strains and three wild mice strains (44), may aid in this effort. Furthermore, while immunity against LM has been shown in inbred mice to be mediated primarily by memory CD8 T cells (37–40), our data indicated that other cell types, including CD4 T cells contribute to immune-mediated protection against LM. Thus, it is possible that differences in the composition or function of other immune cells such as CD4 T cells and B cells, as well as innate immune cells contribute to differences in immune-mediated protection against LM in individual outbred mice. Future studies should examine differences in the memory CD4 T cell and B cell responses following infection in outbred mice as well as differences in the responses of innate immune cells. Additionally, while our depletion studies indicated that cells other than CD8 T cells contribute to protection against re-infection with LM, it is possible that tissue-resident memory CD8 T cells contribute to differences in protection against re-infection seen in individual outbred mice. Determining whether generation of tissue resident memory CD8 T cells is also variable in individual outbred mice, and how these potential differences impact protection against re-infection is an important area of further studies.

Studies in inbred mice have suggested that the quality of memory CD8 T cells present at the time of re-infection impact memory CD8 T cell-mediated protection (13, 27, 31, 35). We found that the phenotype of memory CD8 T cells that persisted following infection differed between individual outbred mice, and that changes in phenotype that occur with time after infection occurred at an unequal rate in individual outbred mice. Future studies should be designed to address how differences in the quality of memory CD8 T cells generated in individual outbred mice impacts their ability to provide protection against re-infection. However, these experiments will be complicated due to the observation that phenotypically different memory CD8 T cells generated following infection in outbred mice appear to correspond with the level of memory generated following infection. Any differences in the protective abilities of phenotypically distinct memory populations could be explained by differences in memory CD8 T cell levels. Additionally, while phenotype has been correlated with function in many instances in inbred mice (26–31), it has yet to be determined if phenotypically distinct memory CD8 T cells generated following vaccination in outbred mice are functionally distinct, and future experiments should be conducted to answer this question. Furthermore, differences in phenotype of memory CD8 T cells resulting from infection in outbred mice presents the opportunity to examine factors influencing the generation of memory CD8 T cells with distinct phenotypes following infection and/or vaccination. However, the genetic makeup of individual Swiss mice is unique, and it will take time and considerable effort to identify genetic differences in individual outbred mice that may influence the phenotypic make-up of memory cells that persist following infection. Studies using collaborative cross mice also may aid in this effort. However, this is a worthwhile endeavor, as understanding the factors influencing phenotype of memory CD8 T cells that persist following infection may aid in the design of vaccines that elicit memory CD8 T cells of appropriate quality to provide protection against unique pathogens.

To summarize, we have found that while the CD8 T cell response to infection develops similarly in inbred and outbred mice, aspects of the CD8 T cell response including magnitude of the effector response, size and phenotype of the resulting memory CD8 T cell pool, and degree of protection provided against re-infection by memory CD8 T cells differ in individual inbred mice. These studies have important implications for the design and assessment of vaccines intended to elicit protective memory CD8 T cells in outbred populations such as humans. Using the surrogate activation marker approach, future studies conducted using genetically diverse mice may prove useful for deciphering the mechanisms governing CD8 T cell homeostasis following infection.

## Ethics Statement

This study was carried out in accordance with the recommendations of University of Iowa Animal Care and Use Committee. The protocol was approved by University of Iowa Animal Care and Use Committee.

## Author Contributions

MM and VB designed experiments. MM performed experiments, analyzed data, and wrote the manuscript. DD and SH helped obtaining some data and analyzing some data and reviewed the final manuscript. JH contributed to the discussion and reviewed the final manuscript. VB contributed to writing and editing of the manuscript.

## Conflict of Interest Statement

The authors declare that the research was conducted in the absence of any commercial or financial relationships that could be construed as a potential conflict of interest.

## Supplementary Material

Figure S1 Clearance of lymphocytic choriomeningitis virus (LCMV) and inflammation elicited following LCMV infection in inbred and outbred mice. **(A)** Experimental design. B6 or Swiss mice were infected with 2 × 10^5^ plaque forming unit (PFU) LCMV-Armstrong. **(B)** Concentration of IFN-α (top) or IFN-γ (bottom) detected in serum of infected B6 or Swiss mice at the indicated hours post infection. Dotted line indicates limit of detection. **(C)** Viral titers per g of spleen (left) or per mL of serum (right) on day 4 following infection. Dotted line indicates limit of detection. NS, not statistically significant; **, statistically significant (*p* < 0.01) as determined by Student’s *t*-test. Representative data from one of two independent experiments with 5 to 20 mice per group.

Figure S2 Proportion of CD8 T cells or antigen-experienced CD8 T cells prior to infection does not correlate with response magnitude in outbred mice. B6 or Swiss mice were infected with 2 × 10^5^ plaque forming unit lymphocytic choriomeningitis virus-Armstrong, and percentage of CD8 T cells among lymphocytes or CD8^lo^/CD11a^hi^ and/or CD44^hi^ cells of gated CD8 T cells among peripheral blood lymphocytes (PBL) were determined before and after infection. **(A)** Left: percentage of CD8 T cells among lymphocytes for B6 or Swiss mice in uninfected animals. Right: percentage of CD8 T cells among lymphocytes prior to challenge infection (*y* axis) relative to CD8^lo^/CD11a^hi^ cells of gated CD8 T cells among PBL at day 8 following infection (*x* axis). **(B)** Left: percentage of CD8^lo^/CD11a^hi^ cells of gated CD8 T cells among PBL prior to challenge infection (*y* axis) relative to CD8^lo^/CD11a^hi^ cells of gated CD8 T cells among PBL at day 8 following infection (*x* axis). Right: percentage of CD44^hi^ cells of gated CD8 T cells among PBL prior to challenge infection (*y* axis) relative to CD8^lo^/CD11a^hi^ cells of gated CD8 T cells among PBL at day 8 following infection (*x* axis). Statistical significance of *R*-squared values based on linear regression analysis.

Figure S3 CD8^hi^/CD11a^lo^ cells retain a naïve phenotype over time in inbred and outbred mice. **(A)** Representative histograms of expression of CD127, CD62L, CD27, and KLRG1 for gated CD8^lo^/CD11a^hi^ cells (top) or CD8^hi^/CD11a^lo^ cells (bottom) for B6 (left), and Swiss (right) mice 200 days after lymphocytic choriomeningitis virus (LCMV) infection. Numbers inside histograms indicate the percentage of cells positive for the indicated marker. **(B)** Percentage of gated CD8^hi^/CD11a^lo^ cells expressing the indicated marker for individual B6 (top) or Swiss (bottom) mice on the indicated day after LCMV infection. Representative data from greater than three independent experiments with at least five mice per group per experiment.

Figure S4 Changes in phenotype following lymphocytic choriomeningitis virus (LCMV) infection occur at different rates in individual outbred mice. **(A)** Swiss mice were infected with 2 × 10^5^ plaque forming unit LCMV-Armstrong. Percentage of CD8^lo^/CD11a^hi^ cells expressing the indicated surface protein on the indicated day after infection for individual mice. **(B)** Left: percentage of CD8^lo^/CD11a^hi^ cells of gated CD8 T cells among peripheral blood lymphocytes for individual Swiss mice on the indicated day after infection. Right: percentage of CD8^lo^/CD11a^hi^ cells expressing the indicated surface protein on day 121 after infection. Red boxed mice indicate Swiss mice that display phenotypic progression at a normal rate compared to B6 mice, while blue boxed mice indicate Swiss mice that display slow or no phenotypic progression compared to B6 mice. **(C)** Percentage of CD8^lo^/CD11a^hi^ cells expressing CD69 at the indicated day after LCMV infection. Representative data from two independent experiments with 10 mice per group per experiment.

Figure S5 Clearance of LM and inflammation elicited following LM infection in inbred and outbred mice. **(A)** Experimental design. B6 or Swiss mice were infected with 5 × 10^6^ colony forming unit (CFU) Att LM. **(B)** Percentage of CD8^lo^/CD11a^hi^ cells of gated CD8 T cells among peripheral blood lymphocytes for B6 or Swiss mice at day 7 post infection. Data are combined from two separate and unrelated experiments. **(C)** Concentration of IFN-γ (top) or IL12 (bottom) detected in serum of infected B6 or Swiss mice at the indicated hours post infection. Dotted line indicates limit of detection. **(D)** Bacterial titers per spleen (left) or per g of liver (right) 48 h following infection. NS, not statistically significant; *, statistically significant (*p* < 0.05); **, statistically significant (*p* < 0.01) as determined by Student’s *t*-test. Representative data from two independent experiments with 10 to 20 mice per group per experiment.
