# Supplementary figures and images for: Revealing the Complexity in CD8 T Cell Responses to Infection in Inbred C57B/6 versus Outbred Swiss Mice
Source: Front Immunol. 2017 Nov 22;8:1527. doi: 10.3389/fimmu.2017.01527 (PMC5702636; doi:10.3389/fimmu.2017.01527)

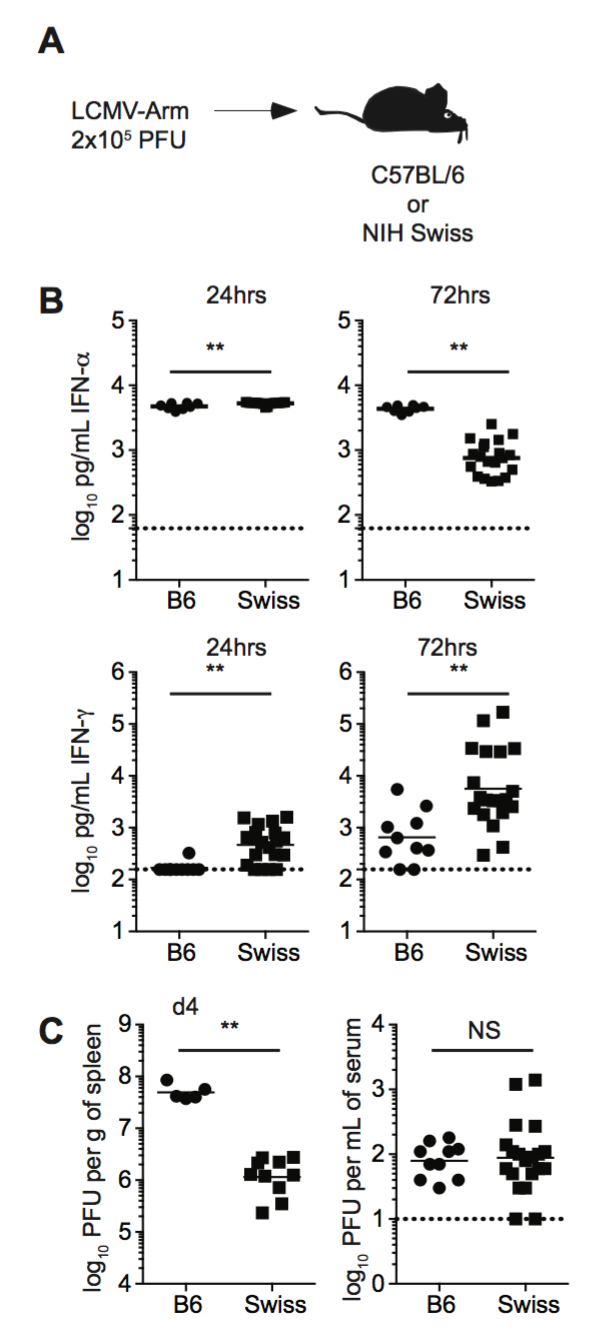

Supplement: Figure S1 — Clearance of lymphocytic choriomeningitis virus (LCMV) and inflammation elicited following LCMV infection in inbred and outbred mice. (A) Experimental design. B6 or Swiss mice were infected with 2 × 10^5^ plaque forming unit (PFU) LCMV-Armstrong. (B) Concentration of IFN-α (top) or IFN-γ (bottom) detected in serum of infected B6 or Swiss mice at the indicated hours post infection. Dotted line indicates limit of detection. (C) Viral titers per g of spleen (left) or per mL of serum (right) on day 4 following infection. Dotted line indicates limit of detection. NS, not statistically significant; **, statistically significant (p < 0.01) as determined by Student’s t-test. Representative data from one of two independent experiments with 5 to 20 mice per group. [file Image_1.tiff]

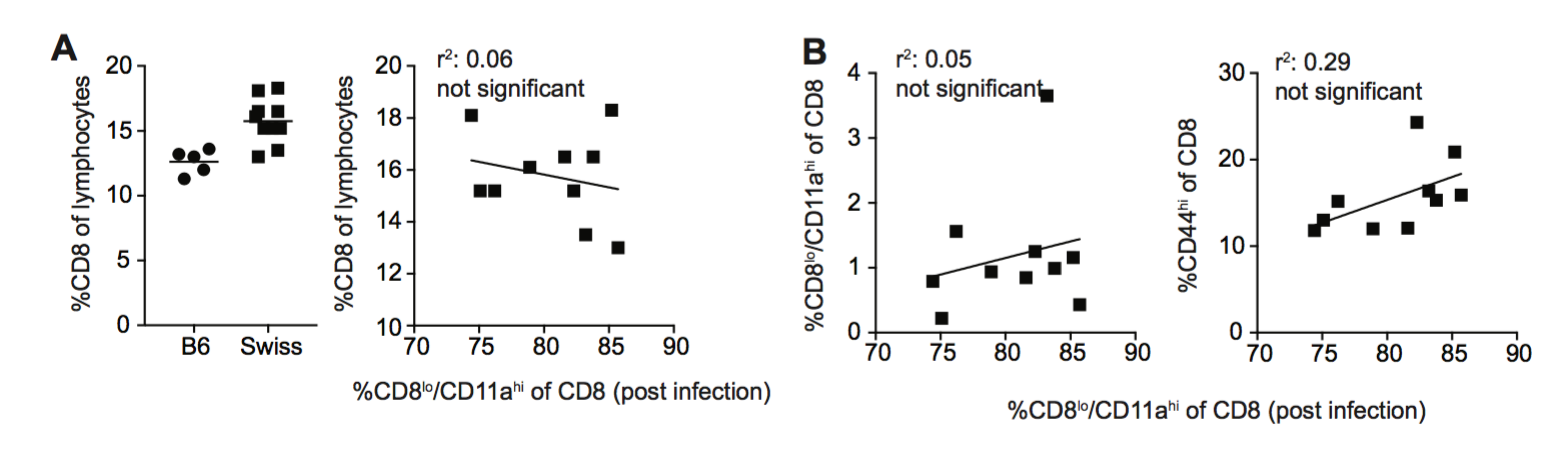

Supplement: Figure S2 — Proportion of CD8 T cells or antigen-experienced CD8 T cells prior to infection does not correlate with response magnitude in outbred mice. B6 or Swiss mice were infected with 2 × 10^5^ plaque forming unit lymphocytic choriomeningitis virus-Armstrong, and percentage of CD8 T cells among lymphocytes or CD8lo/CD11ahi and/or CD44hi cells of gated CD8 T cells among peripheral blood lymphocytes (PBL) were determined before and after infection. (A) Left: percentage of CD8 T cells among lymphocytes for B6 or Swiss mice in uninfected animals. Right: percentage of CD8 T cells among lymphocytes prior to challenge infection (y axis) relative to CD8lo/CD11ahi cells of gated CD8 T cells among PBL at day 8 following infection (x axis). (B) Left: percentage of CD8lo/CD11ahi cells of gated CD8 T cells among PBL prior to challenge infection (y axis) relative to CD8lo/CD11ahi cells of gated CD8 T cells among PBL at day 8 following infection (x axis). Right: percentage of CD44hi cells of gated CD8 T cells among PBL prior to challenge infection (y axis) relative to CD8lo/CD11ahi cells of gated CD8 T cells among PBL at day 8 following infection (x axis). Statistical significance of R-squared values based on linear regression analysis. [file Image_2.tiff]

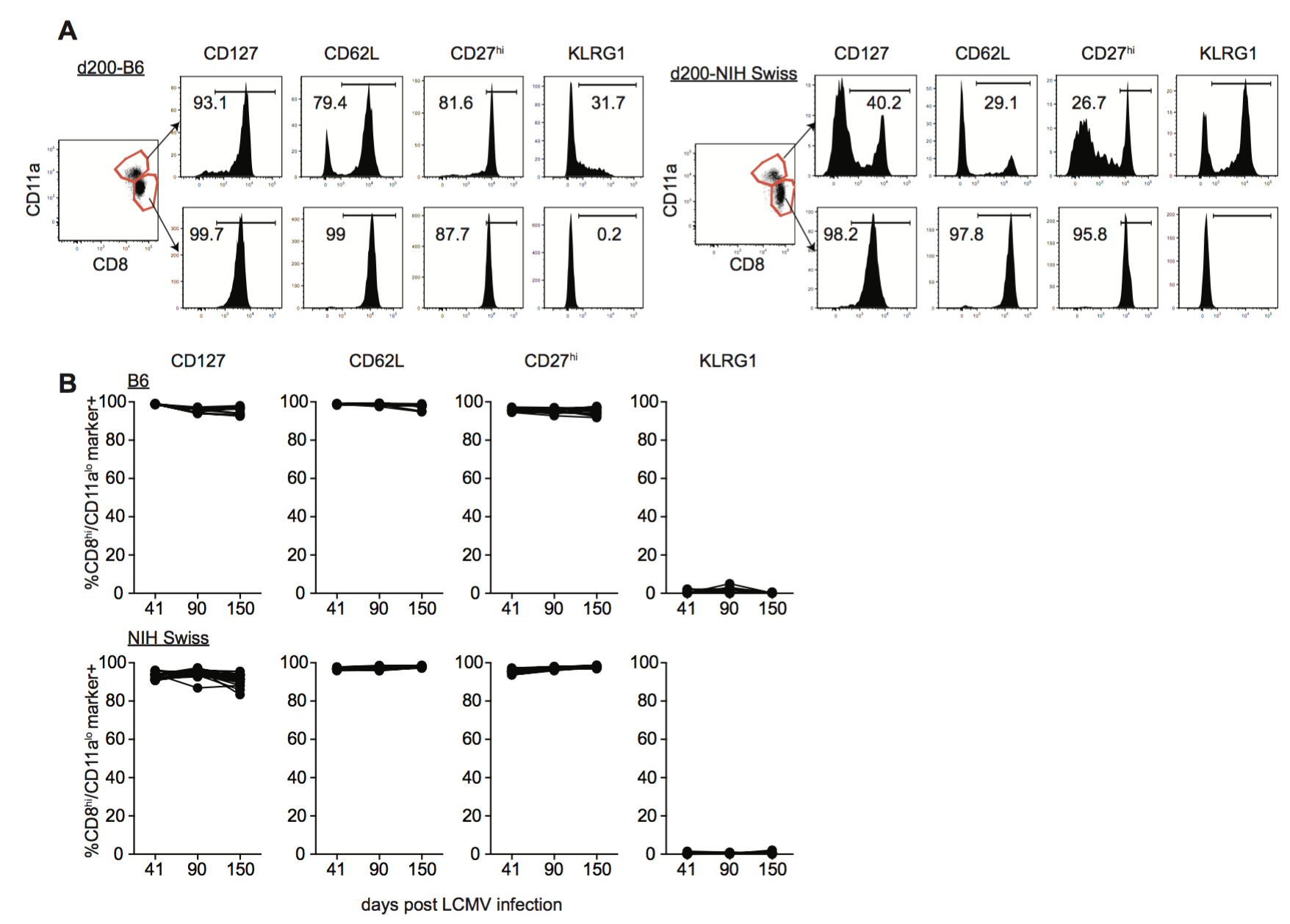

Supplement: Figure S3 — CD8hi/CD11alo cells retain a naïve phenotype over time in inbred and outbred mice. (A) Representative histograms of expression of CD127, CD62L, CD27, and KLRG1 for gated CD8lo/CD11ahi cells (top) or CD8hi/CD11alo cells (bottom) for B6 (left), and Swiss (right) mice 200 days after lymphocytic choriomeningitis virus (LCMV) infection. Numbers inside histograms indicate the percentage of cells positive for the indicated marker. (B) Percentage of gated CD8hi/CD11alo cells expressing the indicated marker for individual B6 (top) or Swiss (bottom) mice on the indicated day after LCMV infection. Representative data from greater than three independent experiments with at least five mice per group per experiment. [file Image_3.tiff]

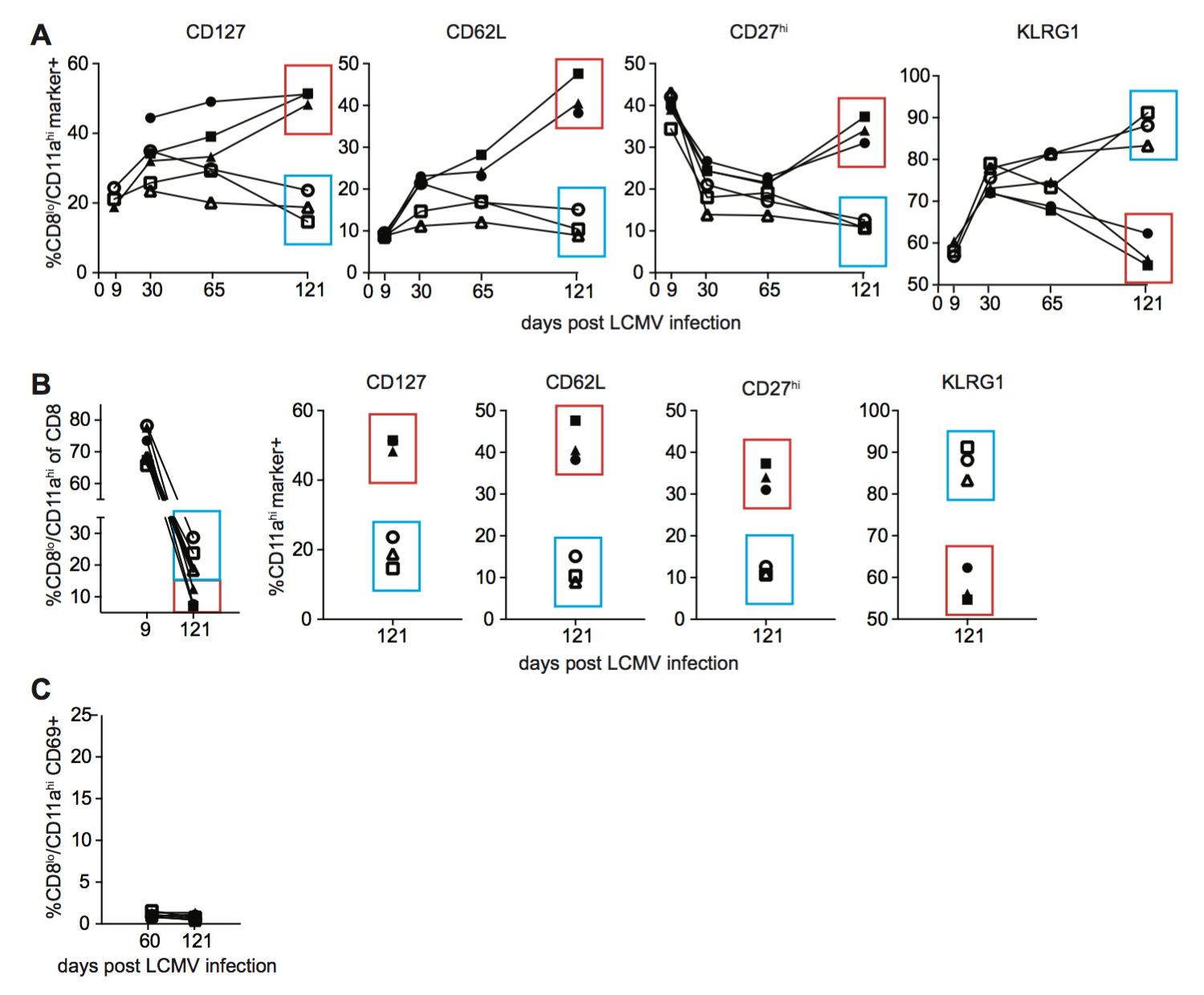

Supplement: Figure S4 — Changes in phenotype following lymphocytic choriomeningitis virus (LCMV) infection occur at different rates in individual outbred mice. (A) Swiss mice were infected with 2 × 10^5^ plaque forming unit LCMV-Armstrong. Percentage of CD8lo/CD11ahi cells expressing the indicated surface protein on the indicated day after infection for individual mice. (B) Left: percentage of CD8lo/CD11ahi cells of gated CD8 T cells among peripheral blood lymphocytes for individual Swiss mice on the indicated day after infection. Right: percentage of CD8lo/CD11ahi cells expressing the indicated surface protein on day 121 after infection. Red boxed mice indicate Swiss mice that display phenotypic progression at a normal rate compared to B6 mice, while blue boxed mice indicate Swiss mice that display slow or no phenotypic progression compared to B6 mice. (C) Percentage of CD8lo/CD11ahi cells expressing CD69 at the indicated day after LCMV infection. Representative data from two independent experiments with 10 mice per group per experiment. [file Image_4.tiff]

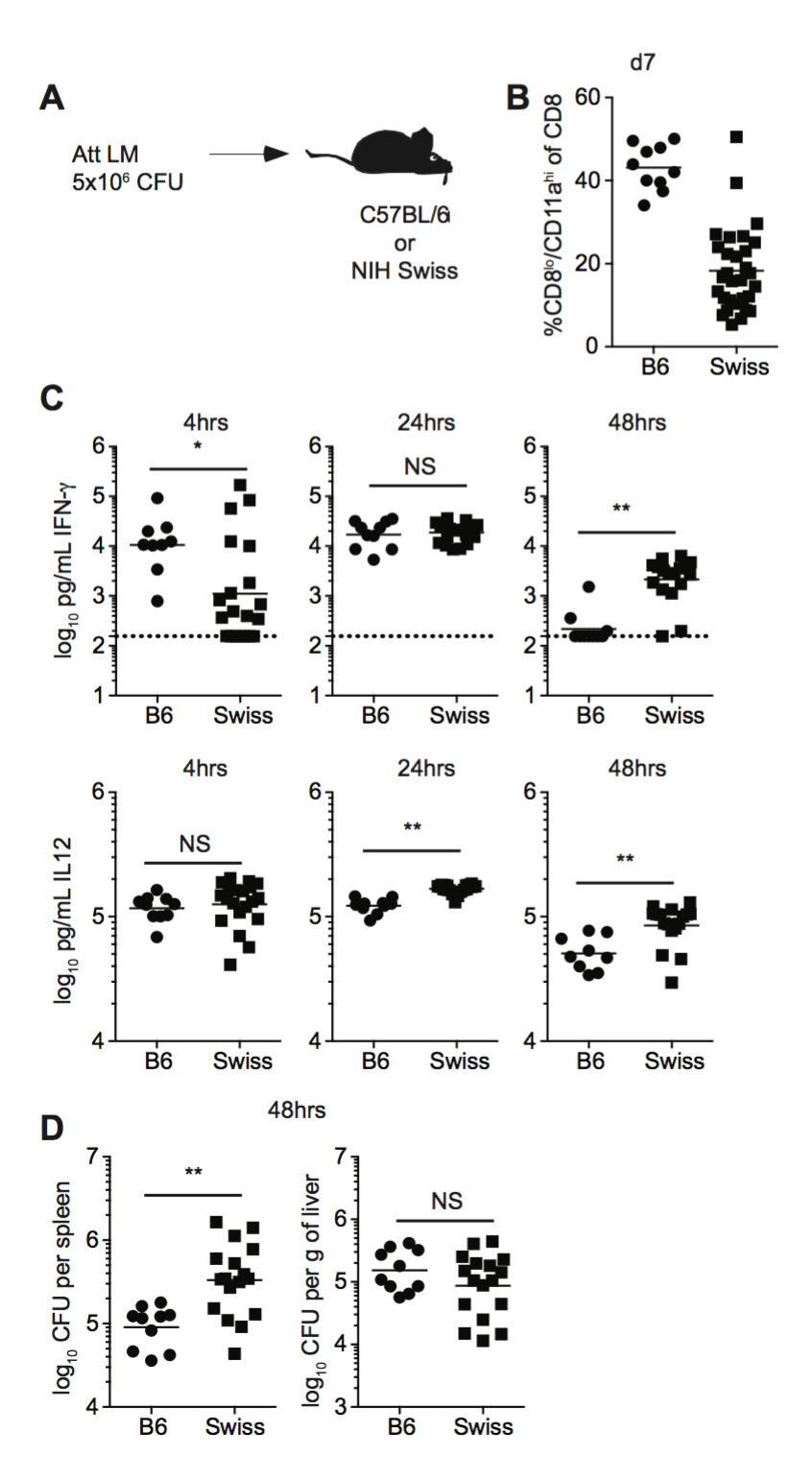

Supplement: Figure S5 — Clearance of LM and inflammation elicited following LM infection in inbred and outbred mice. (A) Experimental design. B6 or Swiss mice were infected with 5 × 10^6^ colony forming unit (CFU) Att LM. (B) Percentage of CD8lo/CD11ahi cells of gated CD8 T cells among peripheral blood lymphocytes for B6 or Swiss mice at day 7 post infection. Data are combined from two separate and unrelated experiments. (C) Concentration of IFN-γ (top) or IL12 (bottom) detected in serum of infected B6 or Swiss mice at the indicated hours post infection. Dotted line indicates limit of detection. (D) Bacterial titers per spleen (left) or per g of liver (right) 48 h following infection. NS, not statistically significant; *, statistically significant (p < 0.05); **, statistically significant (p < 0.01) as determined by Student’s t-test. Representative data from two independent experiments with 10 to 20 mice per group per experiment. [file Image_5.tiff]
